# Blastopore gating mechanism to regulate extracellular fluid excretion

**DOI:** 10.1016/j.isci.2023.106585

**Published:** 2023-04-08

**Authors:** Soichiro Kato, Hidehiko Inomata

**Affiliations:** 1 Laboratory for Axial Pattern Dynamics, Center for Biosystems Dynamics Research, RIKEN, Minatojima-minamimachi, Chuo-ku, Kobe, Hyogo 650-0047, Japan; 2 Laboratory for Developmental Morphogeometry, Center for Biosystems Dynamics Research, RIKEN, Minatojima-minamimachi, Chuo-ku, Kobe, Hyogo 650-0047, Japan; 3 Department of Biological Sciences, Graduate School of Science, Osaka University, Machikaneyama, Toyonaka, Osaka 560-0043, Japan

**Keywords:** Cellular physiology, Developmental biology, Embryology

## Abstract

Fluid uptake and efflux play roles in early embryogenesis as well as in adult homeostasis. Multicellular organisms have two main pathways for fluid movement: cellular-level, such as transcellular and paracellular pathways, and tissue-level, involving muscle contraction. Interestingly, early *Xenopus* embryos with immature functional muscles excrete archenteron fluid via a tissue-level mechanism that opens the blastopore through a gating mechanism that is unclear. Using microelectrodes, we show that the archenteron has a constant fluid pressure and as development progress the blastopore pressure resistance decreases. Combining physical perturbations and imaging analyses, we found that the pushing force exerted by the circumblastoporal collars (CBCs) at the slit periphery regulates pressure resistance. We show that apical constriction at the blastopore dorsoventral ends contributes to this pushing force, and relaxation of ventral constriction causes fluid excretion. These results indicate that actomyosin contraction mediates temporal control of tissue-level blastopore opening and fluid excretion in early *Xenopus* embryos.

## Highlights

• In Xenopus embryos, fluid excretion from the blastopore is temporally regulated
• Apical constriction at the dorsoventral ends drives the pushing force
• The pushing force maintains blastopore closure against archenteron fluid pressure
• The release of the ventral constriction causes opening and fluid excretion

## Graphical abstract

**Figure:**
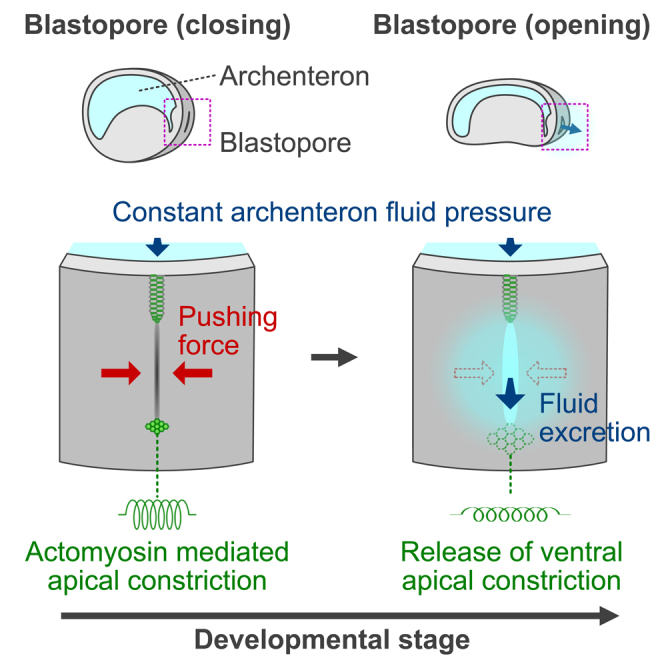

## Introduction

Dynamic control of extracellular fluid via absorption and excretion is a significant component of the maintenance of homeostasis in multicellular organisms. In adult organisms, extracellular fluid is transported through pores and tubes at a microscopic cellular level and at a macroscopic tissue level.^1^ In cellular-level pathways like the paracellular pathway and transcellular pathway, the balance between osmotic and hydraulic pressures determines the direction of fluid transport.^2^^,^^3^ The paracellular pathway transports fluid through small pores, called paracellular pores, in apical tight junctions (TJs) of epithelial and endothelial cells, and various TJ proteins including claudin^4^^,^^5^ and tricellulin^6^ contribute to this fluid transport. Meanwhile, the transcellular pathway takes up fluid through the cell membrane via aquaporins, the water transport channels, and transports fluid through the cell.^7^ The osmotic pressure difference caused by the transport of ions into and out of the cell through co-transporters such as potassium chlorine co-transporter (KCC) and sodium potassium chlorine co-transporter (NKCC) plays an important role in this process.^8^ Cellular-level pathways are utilized in the intestinal tract for water absorption and fluid transfer between vessels and the interstitium, or between vessels and renal tubules in the kidney.^8^ For tissue-level pathways, such as blood flow in blood vessels driven by beating of the heart,^9^ saliva secretion by the contraction of myoepithelium in the salivary gland^10^ and smooth muscle contraction in the bladder during urination,^11^ large volumes of fluid are transported rapidly. Urination also requires the relaxation of the external urethral sphincter,^12^ which is an example of coordinated interactions between the organs to control fluid transport.

Extracellular fluid also plays an important role in early developmental processes.^13^^,^^14^ In early embryos with immature functional muscles, fluid is transported primarily via cellular-level pathways.^14^ Early mouse embryos take in water through the transcellular pathway and form multiple small cavities in the intercellular spaces.^15^^,^^16^ Furthermore, fluid pressure controls the formation and size of the blastocoel cavity in mouse embryos.^17^ Fluid moves through these small cavities in intercellular spaces due to hydraulic pressure differences between the cavities, resulting in the eventual formation of one large blastocyst cavity.^16^ Continued fluid uptake in turn increases the blastocoel size and fluid pressure, resulting in the transient disruption of TJs and fluid excretion at sites where mitotic cell rounding occurred. This excretion then decreases blastocoel fluid pressure to close TJ pores, allowing the enlargement of the cavity by subsequent fluid uptake. The repeated fluid uptake and excretion maintain a constant blastocoel size.^17^ Similar cavity size control is observed in zebrafish embryonic otic vesicles.^18^^,^^19^ These cellular-level microscopic pathways involving pores in TJs, together with transcellular pathways and intercellular spaces, allow the regulation of fluid transport in early embryos that have immature muscles.

Interestingly, early *Xenopus laevis* embryos, which have no functional muscle,^20^^,^^21^ excrete fluid through the blastopore that lies at the opening of the archenteron during the late neurula stage.^22^^,^^23^ This process is termed “collapse of the archenteron.” However, the mechanism that controls blastopore opening at a tissue level is unclear. During gastrulation the blastopore forms at the posterior end of the embryo. Gastrulation begins with the invagination of the dorsal bottle cell that progress laterally to ventrally to form a circular involuting marginal zone (IMZ). Thereafter, the mesendoderm continues to invaginate and the IMZ circumference decreases.^24^ This decrease is largely due to two developmental events: convergent thickening, which reduces the epithelial surface area by cell migration from superficial to deep layers, and convergent extension, which reduces cell numbers by the rearrangement of cells in the plane.^24^^,^^25^^,^^26^ Convergent extension is mainly regulated by actomyosin-mediated contraction associated with Wnt/PCP pathway activation,^27^^,^^28^^,^^29^^,^^30^ whereas interfacial tension between the superficial and deep epithelial layers of the IMZ plays a key role in convergent thickening.^26^ IMZ contractility increases as blastopore formation progress, as shown by force measurements using the deflection of aramide or optical fiber probes.^25^^,^^31^ At the late gastrula stage, mesendoderm involution ceases and the blastopore forms a closed slit surrounded by a thick band of tissue called the circumblastoporal collar (CBC).^32^^,^^33^ Although blastopore formation has been studied in detail, how blastopore opening is regulated in early embryos that have immature muscles is unclear.

To characterize the blastopore gating mechanism, in this study we used microelectrodes to measure archenteron fluid pressure. We demonstrate that archenteron fluid pressure remains constant, but the upper limit of blastopore pressure resistance decreases as development progress. Furthermore, by combining physical perturbation experiments and imaging analysis, we found that the upper limit of pressure resistance is regulated by the force with which the CBCs push against each other. Focusing on actomyosin contraction to mediate this pushing force, we detected the phosphorylation of myosin light chains (pMLC) at the blastopore ventral and dorsal ends during the mid-neurula stage. However, in the late neurula stage just before fluid excretion, pMLC signals at the ventral end of the blastopore were attenuated. Injection of an inhibitor of actomyosin contraction into the ventral end artificially causes blastopore opening and fluid excretion, even in the mid-neurula stage. These results demonstrate that actomyosin contraction in the ventral end of the blastopore defines the upper limit of pressure resistance and that the decrease in pressure resistance opens the blastopore to allow fluid excretion. Our findings show that even early embryos with immature functional muscles can regulate blastopore opening via actomyosin-mediated apical constriction.

## Results

### Laterality defects following removal of archenteron fluid

**Figure 1:**
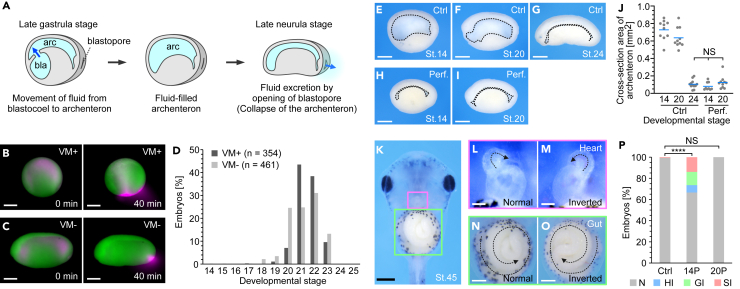
Laterality defects induced by early removal of archenteron fluid (A) Schematic of extracellular fluid transport in *Xenopus* embryos. bla, blastocoel; arc, archenteron. (B and C) Snapshot images from representative time-lapse imaging of fluid excretion in embryos with (B; n = 8) or without (C; n = 6) the vitelline membrane (VM). Cells, green; extracellular fluid, magenta. Scale bars = 500 μm. (D) Measurement of fluid excretion stages. VM+, n = 354; VM-, n = 461. (E-I) Sagittal section of control embryos (Ctrl; E-G) and perforated embryos (Perf.; H, I). The black dotted line outlines the archenteron. Scale bars = 500 μm. (J) Cross-section area of the archenteron in (E-I): Control embryos at stage 14 (n = 10), stage 20 (n = 10), and stage 24 (n = 12). Perforated embryos at stage 14 (n = 9) and stage 20 (n = 10). Blue lines, mean. (K-O) Ventral view of embryos at stage 45. Magenta and green squares indicate the heart and gut, respectively (K). Magnified view of normal (L) and inverted (M) heart. Magnified view of normal (N) and inverted (O) gut. Scale bars = 500 μm (K), 100 μm (L, M), 300 μm (N, O). (P) Ratio of normal and laterality defects. Control embryos (Ctrl; n = 176). Embryos perforated at stage 14 (14P; n = 48) or stage 20 (20P; n = 59). N, normal; HI, heart inverted; GI , gut inverted; SI, situs inversus. ∗∗∗∗p < 0.0001. n = number of animals used. See also Video S1.

During the early development of *Xenopus laevis*, extracellular fluid in the blastocoel moves into the archenteron. The archenteron fluid is then excreted from the embryo through the blastopore, a process called “collapse of the archenteron” that occurs via a mechanism that has not been fully characterized (Figure 1A).^23^ To examine the temporal regulation of fluid excretion in *Xenopus* embryos, we injected a fluorescent dye, 10 kDa Alexa Fluor 568-dextran, into the archenteron at stage 14, and analyzed the timing of fluid excretion in the presence and absence of the vitelline membrane (a glycoprotein-rich membrane that covers the embryo until the tail bud stage). We saw the excretion of archenteron fluid from the embryo after stage 20 whether or not the vitelline membrane was present (Figures 1B-1D, Video S1), suggesting that the timing of fluid excretion is limited to a specific period.

Video S1. Archenteron fluid excretion through the open blastopore, related to Figure 1 First part: Time-lapse images of the lateral view of the embryo with (upper) and without (lower) the vitelline membrane during archenteron fluid excretion. The time at which the fluid excreted was set to t = 0 min. Green, cells; magenta, extracellular fluid. Second part: Time-lapse image of the blastopore during archenteron fluid excretion. The vitelline membrane was removed prior to time-lapse imaging. The time at which the fluid excreted was set to t = 0 min. Green, cells; magenta, extracellular fluid.

We next artificially removed the archenteron fluid before stage 20 and examined the effects on development. When the anterior part of the archenteron was punctured with a glass needle at stage 14 (before excretion) or stage 20 (just before excretion), most of the fluid was excreted from the opening, and strong archenteron contraction was observed, as in stage 24 embryos (i.e., after the collapse of the archenteron) (Figures 1E-1J). In *Xenopus* embryos, the unidirectional fluid flow induced by rotating cilia present in the gastrocoel roof plate (GRP) plays a central role in the determination of the left-right axis between stages 15 and 19.^34^ Therefore, early removal of archenteron fluid could cause laterality defects. To examine this possibility, the direction of heart and gut looping in embryos from which fluid was removed was analyzed at stage 45. The embryos from which fluid was removed at stage 14 showed an inversion of the left-right axis, but no significant effects were observed at stage 20 (Figures 1K-1P). These results suggest that the temporal regulation of fluid excretion through the blastopore is important in left-right axis formation.

### Opening of the blastopore due to decreased pressure resistance

Extracellular fluid is directionally excreted from the blastopore to the medium (Video S1). As such, the fluid pressure in the archenteron is likely higher than that outside the embryo. Consistent with this prediction, 1 μm fluorescent beads injected into the archenteron were excreted through a perforated pore to the outside of the embryo (Video S2). These results also suggest that the positive pressure in the archenteron persists until most of the fluid is excreted from the embryo (Figures 1E-1I). Therefore, we hypothesized that an increase in archenteron fluid pressure as development progress would promote blastopore opening and fluid excretion. To measure fluid pressure in the archenteron, we used glass microelectrodes (900A system) filled with 3M NaCl solution that have low electrical resistance (Figure S1A). When the tip of the microelectrode (7.4 ± 0.8 μm, see STAR Methods for details) is inserted into the archenteron where the pressure is presumed to be high, extracellular fluid having a low salt concentration and high electrical resistance flows into the microelectrode. The system detects an increase in electrical resistance and pushes back the incoming fluid into the archenteron by applying counterpressure in the microelectrode. The 900A system maintains a constant electrical resistance (servo-null system) by repeating a series of controls at very short intervals (approximately 10 ms), such that the counter pressure is the same value as the fluid pressure. Increasing the depth of the microelectrode at 0.1 mm intervals was associated with a linear change in water pressure (Figure S1B). The change in water depth during insertion into the embryo was corrected by attaching a dial gauge to the manipulator, which can measure the distance of the microelectrode tip movements (Figures S1C and S1D); the pressure measurements are the corrected results.

**Figure 2:**
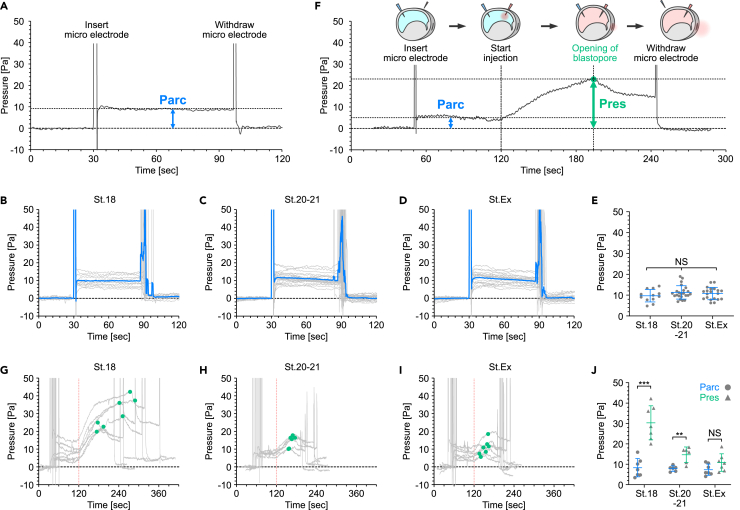
Excretion of extracellular fluid induced by decreases in the upper limit of pressure resistance (A) Representative measurement data of pressure in the archenteron (Parc) at stage 18. (B-D) Pressure measurement at stage 18 (St.18; n = 12) (B), stage 20-21 (St.20-21; n = 23) (C), and excretion stage (St.Ex; n = 20) (D). Blue line; mean curve. (E) Parc at different stages. Blue line; mean ± SD. (F) Representative data in the measurement of the upper limit of the pressure resistance (Pres) at stage 18. (G-I) Pres at stage 18 (St.18; n = 7) (G), stage 20-21 (St.20-21; n = 6) (H), and excretion stage (St.Ex, n = 7) (I). Continuous phenol red injection began at t = 120 s. Green dots, Pres at which fluids were excreted. (J) Pres at different stages. Blue line, mean ± SD (Parc); green line, mean ± SD (Pres). ∗∗p = 0.0043, ∗∗∗p = 0.0006. n = number of animals used. See also Figures S1-S3 and Videos S2, S3,and S4.

Video S2. Ejection of fluorescent beads from a perforation in the archenteron, related to Figure 2 Time-lapse image of fluorescent beads ejected from a perforation in the archenteron.

This system measures pressure via the electrical resistance of the fluid such that the salt concentration of the fluid flowing into the microelectrode affects the measured value. Previous reports indicate that the salt concentration of archenteron fluid is higher than that of 0.1x Barth’s medium used for *Xenopus* embryos, suggesting that changes in concentrations during measurement must be considered.^23^ To verify this, archenteron fluid recovered from stages 18 and 20 embryos was loaded into glass capillaries for the measurement of electrical resistance, which was found to be between 5.5 and 6.0 MΩ (Figures S2A and S2C). A calibration curve with electrical resistance as a function of salt concentration in which only the NaCl concentration of the 0.1x Barth’s medium was changed revealed that the electrical resistance of the archenteron fluid is approximately equal to 0.1x Barth’s medium containing 34.8-38.7 mM NaCl (Figure S2B). Therefore, we prepared Arc-buffer, 0.1x Barth’s medium containing 36.7 mM NaCl, with an electrical resistance similar to that of the archenteron fluid (Figure S2C). The change in the pressure measurement value without depth change was compared between the point when the medium was switched from Arc-buffer to Arc-buffer (i.e., no change) or buffer having the same electrical resistance as archenteron fluid at stage 18 or stage 20; all values were the same, ∼0 (Figure S2D). This result suggests that fluid electrical resistance has no effect on the values measured at each stage. Embryos cultured in Arc-buffer also excreted fluid at the same time as embryos cultured in 0.1x Barth’s medium (Figure S2E). These results show that the use of Arc-buffer allows the measurement of fluid pressure without considering changes in concentration during measurement and the possible effects on fluid excretion.

Using Arc-buffer, we examined whether the archenteron pressure (Parc) increases due to developmental progression by measuring the pressure at three stages: stage 18 (before excretion), stage 20-21 (just before excretion), and the stage during excretion (St.Ex; embryos from stage 20 onward) (Figure 2A). Intriguingly, at all three stages, we observed no significant difference in fluid pressure, which remained around 10 Pa (Figures 2B-2E). When the microelectrode was inserted into embryos in which the archenteron fluid was fluorescently labeled, no obvious leakage of fluid was observed upon insertion (Video S3).

Video S3. No fluid leakage associated with microelectrode insertion, related to Figure 2 Time-lapse image of a lateral view of an embryo. A microelectrode was inserted into the embryo having fluorescently labeled archenteron fluid. The time at which the microelectrode was inserted into the archenteron was set to t = 0.

We next hypothesized that the upper limit of pressure resistance (Pres), at which the blastopore maintained closure against the hydrostatic pressure of the archenteron, may decrease due to the progression of developmental stages and could fall below the fluid pressure in the archenteron, resulting in fluid excretion. To examine the upper limit of blastopore pressure resistance, we first inserted a pressure-measuring microelectrode into the archenteron and analyzed fluid pressure (Parc) (Figures 2F, S3A, S3E, and Video S4). Thereafter, fluid pressure was artificially increased by consecutively injecting (5 nL/s) Arc-buffer containing phenol red (Arc-PR-buffer), which has the same electrical resistance as Arc-buffer (Figure S2C), into the archenteron (Figures 2F and S3B). We measured Pres at which the blastopore opened and excreted fluid (Figures 2F and S3C-S3E). Pres was around 30 Pa at stage 18 but by stage 20-21 the Pres decreased to around 15 Pa (Figures 2G, 2H, and 2J). During the excretion stage (St. Ex), the Pres value was essentially the same as that for Parc (Figures 2I and 2J). Examination of how buffer injection rate affected Pres in stage 18 embryos revealed no significant differences in Pres at 1.25, 2.5, and 5 nL/s (Figure S3F). This result suggests that the injection rate used in this study is slow enough so as not to affect the viscosity of the tissue, or that the viscosity itself is small. Taken together, these results show that a constant pressure, Parc, is applied to the blastopore and that extracellular fluid is excreted through the blastopore as the Pres decreases and reaches the Parc.

Video S4. Measurement of the upper limit of pressure resistance, related to Figure 2 Time-lapse image of Arc-PR-buffer being injected into the archenteron while measuring fluid pressure. The time at which the Arc-PR-buffer was injected into the archenteron was set to t = 0.

### Adhesion between circumblastoporal collars does not contribute to blastopore closure

**Figure 3:**
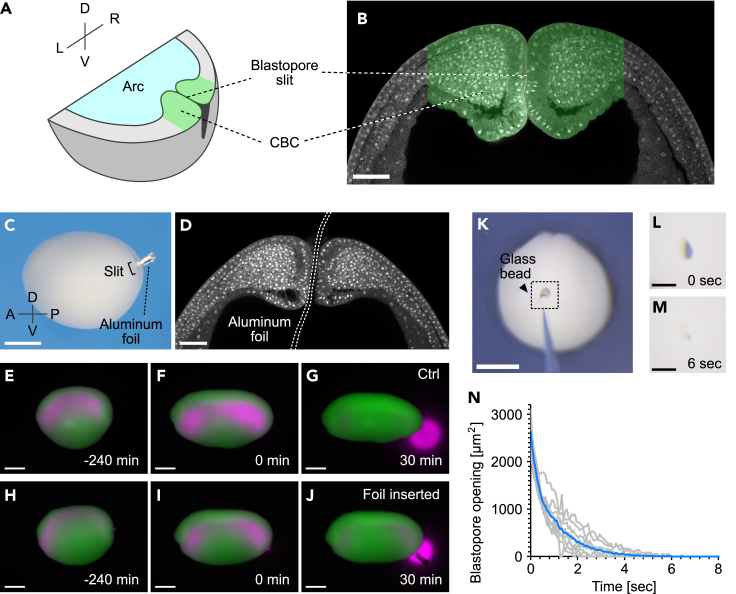
Effect of adhesion between CBCs on blastopore closure (A) Blastopore and CBC structure. (B) Coronal section of the blastopore cleared with benzyl alcohol/benzyl benzoate (BABB) solution. Green area, CBC. Scale bar = 100 μm. (C) Embryos with aluminum foil inserted into the blastopore. Scale bar = 500 μm. (D) Coronal section image of the blastopore with aluminum foil inserted and cleared with BABB. White dotted line outlines the aluminum foil. Scale bar = 100 μm. (E-J) Snapshot images from representative time-lapse imaging of fluid excretion in control (E-G; n = 6) and aluminum foil-inserted embryo (H-J; n = 6). Green, cells; magenta, archenteron fluid. Initiation of fluid excretion was set as 0 min. Scale bars = 500 μm. (K) Representative image of an embryo with glass bead inserted. (L-M) Magnified view of the dotted area in (K) immediately after removing the bead (L) and 6 s later (M). (N) Change over time in blastopore opening area (n = 9). Scale bars = 500 μm (K), 100 μm (L, M). n = number of animals used. See also Figure S4 and Videos S5 and S6.

We next focused on the regulatory mechanism of blastopore pressure resistance. The blastopore is formed at the posterior end of the embryo, where mesoderm invagination occurs, and has a slit-like gap extending in the dorsoventral direction (Figure 3A). The slit periphery is surrounded by a thick band of tissue called the circumblastoporal collar (CBC) (Figure 3B). Transmission electron microscopy analysis (TEM) of the blastopore revealed irregular wide gaps (10-700 nm) between CBCs, which partially formed adherent junction-like close contacts (Figures S4A-S4C).^35^ Thus, blastopore closure could be regulated by physical adhesion between CBCs mediated by adhesion molecules or the ECM. To address this possibility, a 100 μm long x 5 μm high piece of aluminum foil was inserted into the blastopore to physically break the adhesion (Figures 3C and 3D). Because the length of the slit long axis was approximately 140 μm from stage 14 to the excretion stage (Figure S4D), adhesion between CBCs was effectively separated by this piece of aluminum foil. However, the insertion of the strip did not induce fluid excretion, and embryos with the inserted strip excreted fluid by the collapse of the archenteron at the same time as the control embryos (Figures 3E-3J, Video S5). Furthermore, when the slit was artificially opened by inserting a glass bead, the blastopore closed within a few seconds after bead removal (Figures 3K-3N, Video S6). These results indicate that physical adhesion between CBCs is not the main mechanism for maintaining blastopore closure.

Video S5. Fluid excretion in an aluminum foil-inserted embryo, related to Figure 3 Time-lapse images of the lateral view of a normal (upper) and aluminum foil-inserted (lower) embryo. The time at which the fluid was excreted was set to t = 0. Green, cells; magenta, extracellular fluid.

Video S6. Blastopore closure after glass bead insertion, related to Figure 3 Time-lapse image of the blastopore with the glass bead inserted. The time when the glass bead was removed was set to t = 0 s.

### Regulation of blastopore pressure resistance via pushing force

**Figure 4:**
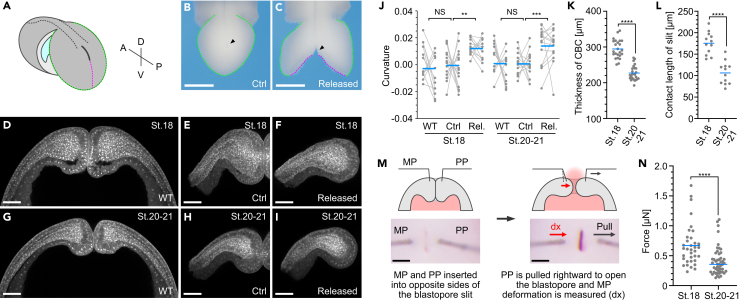
Decrease in pushing force during fluid excretion (A) Schematic of the experiment to release pushing force. The circumference (green line) and ventral side (magenta line) of the blastopore were excised, leaving the dorsal region. (B and C) Control embryos with uncut ventral end (B), and embryos with pushing force released (C). Arrowhead, blastopore. Scale bars = 500 μm. (D-I) Coronal section of the blastopore cleared with BABB. Wild-type embryos at stage 18 (D) and 20-21 (G). Control embryos with uncut ventral end at stage 18 (E) and 20-21 (H). Embryos with pushing force released at stage 18 (F) and 20-21 (I). Scale bars = 100 μm. (J) Curvature of the CBC contact surface. Left and right columns show CBC curvature on the left and right sides, respectively. Gray lines correspond to the CBC of the same embryo. WT, wild-type embryo (stage 18, n = 12; stage 20-21, n = 12); Ctrl, control embryos (stage 18, n = 16; stage 20-21, n = 13); Rel., pushing force released embryo (stage 18, n = 13; stage 20-21, n = 14). ∗∗p = 0.0018, ∗∗∗p = 0.0007. (K) Thickness of CBC at stage 18 (N = 24 CBCs from 12 embryos) and stage 20-21 (N = 24 CBCs from 12 embryos). Blue line; mean. ∗∗∗∗p < 0.0001. (L) Contact length of the slit at stage 18 (n = 12) and stage 20-21 (n = 12). Blue line; mean. ∗∗∗∗p < 0.0001. (M) Schematic of force measurement. MP, Measurement probe; PP, Pulling probe. Scale bars = 100 μm. (N) Force required to open the blastopore at stage 18 (n = 35) and stage 20-21 (n = 49). Blue line; mean. ∗∗∗∗p < 0.0001. n = number of animals used. N = other data points. See also Figures S5 and S6 and Video S7.

The closure of the blastopore shortly after bead removal (Figures 3K-3N) suggests that blastopore pressure resistance may be controlled by the force of CBCs pushing against each other (termed “pushing force”). To investigate whether the formation of the long contact surface observed during blastopore closure is due to a pushing force, we cut the ventral side of the slit to relieve this force. Specifically, the archenteron periphery was excised at stages 18 and 20-21, leaving the dorsal side (Figures 4A and 4B). Thereafter, the ventral side of the slit was cut to release the pushing force, and the embryo was fixed within 1 min (Figure 4C). In the uncut piece of tissue, a long contact surface of CBCs was observed as in the wild-type embryo; however, in the cut tissue piece, the contact surface became convex (Figures 4D-4I). A more detailed analysis of the curvature on both sides of the contact surface showed that in wild-type embryos and ventral uncut tissue pieces, concave and convex surfaces or flat surfaces were in contact, with an average value of ∼0 (Figures 4J and S5A-S5G). However, in the ventral cut tissue pieces a positive curvature with convex surfaces dominated (Figure 4J). Furthermore, when the blastopore was fixed by directly injecting fixative solution into the archenteron at the excretion stage, the CBC had a convex shape similar to that of the incised embryos (Figures S5H and S5I). These results suggest that CBCs with convex surfaces push against each other at the contact surface, resulting in deformation.

We next analyzed the stage-dependent shape changes of the CBC using the CBC thickness and the contact surface length as an index. The CBC thickness was decreased at stage 20-21 relative to that at stage 18 (Figures 4D, 4G, and 4K) and the contact surface showed a similar reduction (Figures 4D, 4G, and 4L). A large pushing force is expected to strongly compact the CBC, increasing its thickness and contact surface; therefore, these results suggest the attenuation of the pushing force with the developmental stage.

To examine the pushing force of the CBC more directly, we inserted 30 μm-thick tungsten wire probes (Figure S6A) into both sides of the slit, and measured the force required to open the blastopore (Figure 4M). Only the pulling probe (PP) was moved horizontally, and the force was calculated based on strain on the measuring probe (MP). To examine the relationship between strain and stress, a calibration curve was prepared by repeatedly adding 20 nL of silicon oil to the tip of the measuring probe placed horizontally, which showed linearity between ∼0 and 4 μN (Figures S6B, S6C, Video S7). Using these probes, the force required to open the blastopore decreased from 0.67 μN at stage 18 to 0.4 μN at stage 20-21 (Figure 4N, Video S7). Further, by inserting both probes into one side of the slit, we measured the force required to stretch the CBC in the direction of the slit long axis. No significant differences were observed between stages 18 and 20-21 (Figures S6D-S6F). This result indicates that the stiffness of the CBC is nearly constant during both stages, or that the change in stiffness is too small to detect with the tungsten wire.

Video S7. Measurement of force required to open the blastopore, related to Figure 4 First part: Calibration of force measurement probe. Silicone oil was repeatedly added in 20 nL volumes to the tip of the probe (right) up to a total volume of 400 nL. Second part: Tungsten wire was used to measure force required to open the blastopore at stage 18 (upper) and stage 20-21 (lower). The archenteron fluid was pre-labeled with phenol red.

These results show that the upper limit of pressure resistance of the blastopore is mainly controlled by the pushing force of CBCs and that a decrease in pushing force may open the blastopores to allow fluid excretion.

### Regulation of pushing force via actomyosin contraction

**Figure 5:**
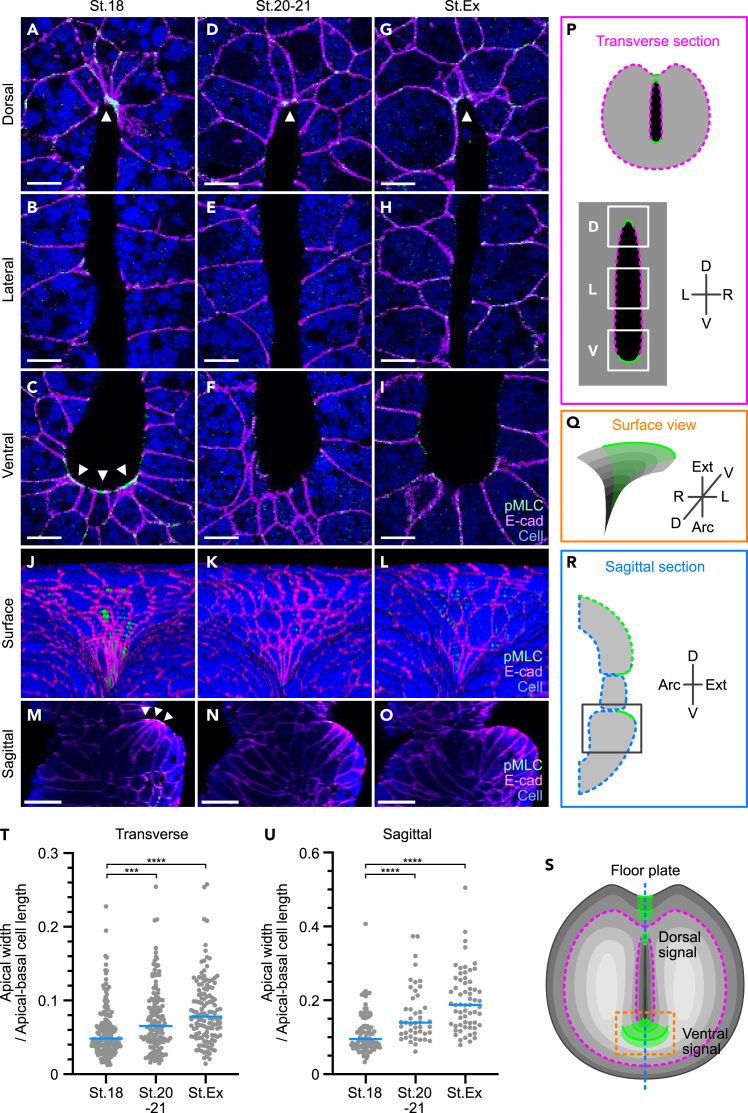
Decrease in ventral actomyosin contraction during blastopore opening (A-O) Immunostaining of the blastopore using anti-pMLC and anti-E-cadherin antibodies. Cells were labeled by the injection of 10 kDa Alexa Fluor 647-dextran. Representative transverse section images of the blastopore cleared with BABB at stage 18 (A-C), stage 20-21 (D-F), and excretion stage (G-I). Representative images of the surface (J-L) and sagittal section (M-O) in the ventral end of the blastopore reconstructed from continuous optical sections. Green, pMLC; magenta, E-cadherin; blue, cells; arrowheads, pMLC signals. Scale bars = 10 μm (A-I), 50 μm (M-O). (P-R) Schematic of transverse section (P), ventral surface (Q), and sagittal section (R) of the blastopore. Green, pMLC signals; Magenta, and blue dotted lines correspond to that of the summary figure S. (S) Posterior view of the blastopore. (P) and (R) are schematics of the region transected by the magenta and blue dotted lines in (S), respectively. (N) corresponds to the orange square in (S). (T and U) Ratio of apical width to apical-basal length of epithelial cells at the ventral end of the blastopore. Transverse section; stage 18 (N = 165 cells from 7 embryos), stage 20-21 (N = 137 cells from 5 embryos), excretion stage, (N = 129 cells from 6 embryos). Sagittal section; stage 18 (N = 97 cells from 7 embryos), stage 20-21 (N = 46 cells from 5 embryos), excretion stage (N = 62 cells from 6 embryos). Blue lines, median. ∗∗∗p = 0.0006, ∗∗∗∗p < 0.0001.

We next focused on the mechanism to generate pushing force, which resists the positive pressure of the archenteron fluid and maintains blastopore closure. During development, actomyosin, which comprises actin fibers and myosin, causes the apical constriction of epithelial cells upon myosin light chain (pMLC) phosphorylation. Coordinate apical constriction of multiple cells drives large forces that induce changes in tissue shape.^36^^,^^37^ For instance, in *Xenopus* embryos, bottle cells during gastrulation^38^ and floor plate cells during neural tube formation induce global tissue shape changes through apical constriction.^39^ We analyzed whether actomyosin contraction was observed in the blastopore periphery using immunofluorescence staining of pMLC. The staining intensity was strong in the dorsal and ventral ends of the blastopore in the transverse section of stage 18 embryos (Figures 5A-5C, 5P and 5S). However, at stage 20-21 (just before excretion) and during the excretion stage, pMLC signals were maintained in the dorsal end of the blastopore, whereas ventral signals were attenuated or disappeared (Figures 5D-5I, 5P, and 5S). To examine the ventral pMLC signals in more detail, a three-dimensional structure of the blastopore was reconstructed using transverse sections. Observation of the tissue surface in the ventral blastopore revealed that pMLC signals extended throughout the ventral side at stage 18, but were attenuated at stage 20-21 and in the excretion stage (Figures 5J-5L, 5Q, and 5S). Sagittal sections of the reconstructed image further revealed that ventral pMLC signals were detected on the blastopore surface but not inside the embryo (Figures 5M-5O, 5R, and 5S).

We next investigated whether apical constriction was observed in the pMLC-positive tissue. When cell shape was analyzed by fluorescent immunostaining with an anti-E-cadherin antibody, apical surface constriction was detected in the ventral side of the blastopore at stage 18 (Figure 5J). However, the contraction was resolved and the apical surface area increased at stages 20-21 and the excretion stage (Figures 5K and 5L). To quantitatively analyze apical constriction, the ratio of the apical to the apical-basal length of ventral cells was measured using coronal and sagittal sections of the reconstructed image (Figures 5T and 5U). In both slices, apical constrictions were weaker in the later stages. These results suggest that actomyosin-dependent apical constriction in the dorsal and ventral ends of the blastopore contributes to the pushing force, and that a decrease in ventral contraction induces blastopore opening.

### Opening of the blastopore by the inhibition of actomyosin contraction

**Figure 6:**
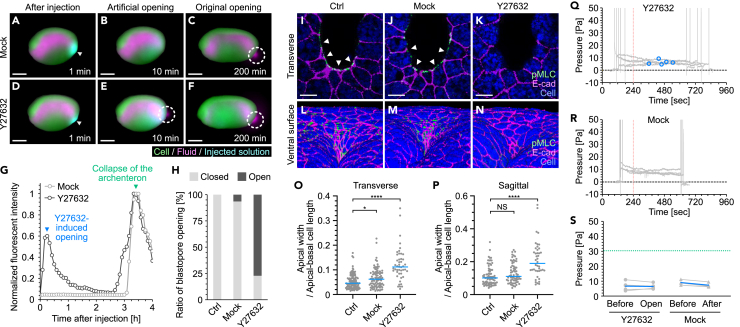
Opening of the blastopore by Y27632 injection (A-F) Snapshot of representative time-lapse images of mock- (A-C) and Y27632-injected embryos (D-F). Time indicates the elapsed time after Y27632 injection. Arrowhead, blastopore; dotted circle, fluid excretion. Green, cells; magenta, archenteron fluid; cyan, Alexa 488 hydrazide. Scale bars = 500 μm. (G) Representative excretion process of the archenteron fluid after the injection of Mock (light gray) or Y27632 (dark gray). Blue arrowhead, Y27632-induced opening of the blastopore; green arrowhead, collapse of the archenteron. (H) Ratio of blastopore opening within 10 min of Mock (n = 2/31) or Y27632 (n = 27/35) injection. Ctrl, embryos with no injection (n = 0/28). (I-N) Immunostaining images of embryos fixed 10 min after Y27632 injection. Representative transverse section (I-K) and reconstructed ventral surface (L-N) of the blastopore. Ctrl, uninjected embryos; Mock, mock-injected embryos; Y27632, Y27632-injected embryos. Green, pMLC; magenta, E-cadherin; blue, cells; arrowheads, pMLC signals. Scale bars = 10 μm (I-K). (O and P) Ratio of apical width to apical-basal length of epithelial cells in the ventral end of the blastopore at stage 18. Transverse section; Ctrl (N = 110 cells from 4 embryos), Mock (N = 90 cells from 4 embryos), Y27632 (N = 46 cells from 4 embryos). Sagittal section; Ctrl (N = 76 cells from 4 embryos), Mock (N = 67 cells from 4 embryos), Y27632 (N = 42 cells from 4 embryos). Blue lines, median. ∗p = 0.0406, ∗∗∗∗p < 0.0001. (Q and R) Pressure in the archenteron after mock (n = 4) or Y27632 (n = 5) injection. The mock or Y27632 injections were made at t = 240. Blue circle, pressure when the blastopore opened. (S) Pressure in the archenteron after Mock or Y27632 injection. Before, pressure before injection; open (Y27632), pressure upon opening of the blastopore; after (Mock), pressure in the mock embryo at the average opening time of Y27632-injected embryo. Blue lines, mean; green line, upper limit of pressure resistance of the blastopore at stage 18 (refer to Figure 2J). n = number of animals used. N = other data points. See also Figure S7 and Video S8.

To investigate the relationship between actomyosin activity and the blastopore gating mechanism, we examined whether suppression of ventral actomyosin activity at stage 18 (before excretion) causes blastopore opening. Y27632 inhibits Rho-kinase, which is involved in myosin phosphorylation, and in turn inhibits apical constriction.^40^ A glass needle was inserted into the blastopore ventral region and a small amount (1 nL) of Y27632 was injected into the tissue. When a fluorescent dye, Alexa 488 hydrazide (MW = 570.48), was co-injected to confirm the injection site of Y27632 (MW = 320.26), it diffused within the tissue and its fluorescence intensity was attenuated (Video S8). This result suggests that, immediately after injection, a high concentration of Y27632 is present in the blastopore ventral region, but it then diffuses and is diluted. Consistent with this assumption, time-lapse imaging following the injection of Y27632 into the blastopore ventral region showed the excretion of extracellular fluid within 10 min, after which the blastopore closed again (Figures 6D, 6E and 6H, Video S8). Interestingly, at the late neurula stage, Y27632-injected embryos again excreted fluid via the collapse of the archenteron (Figures 6F and 6G). In contrast, in embryos with the mock injection of buffer, only fluid excretion associated with the collapse of the archenteron was observed (Figures 6A-6C). At the tadpole stage, Y27632-injected embryos showed no obvious developmental abnormalities (Figures S7A-S7C). In addition, no significant enlargement of the archenteron associated with the relaxation of apical constriction was observed with the Y27632 injection (Figures S7D-S7G). Together, these results indicate that Y27632 inhibits the pushing force and drives blastopore opening only for a short time, and blastopore function and embryonic development are normal.

Video S8. Early fluid excretion by Y27632 injection, related to Figure 6 Time-lapse images of a lateral view of mock- (upper) and Y27632-injected (lower) embryos. The time at which the mock or Y27632 injection into the ventral end of the blastopore occurred was set to t = 0. Green, cells; magenta, extracellular fluid.

We next examined the effect of Y27632 injection on pMLC using immunostaining. Following Y27632 injection into the blastopore ventral region at stage 18, pMLC signals in the ventral region disappeared, and the effects on the dorsal region were insignificant (Figures 6K and S7J). In contrast, in embryos with the mock injection of buffer, the pMLC signal remained on the ventral side of the blastopore (Figures 6I, 6J, S7H, and S7I). When Y27632 was injected into the dorsal region of the blastopore, the dorsal pMLC signal disappeared and the ventral signal was weakened (Figures S7K, S7L, S7O, and S7P). This may be because the dorsal pMLC signal was higher and less likely to be suppressed than the ventral pMLC signal. When equal amounts of Y27632 were directly injected into the archenteron, no effects on pMLC signals were observed, indicating that Y27632 was diluted in the archenteron fluid (Figures S7M, S7N, S7Q, and S7R). These results suggest that ventral side injection of Y27632 transiently and locally suppresses actomyosin contraction in the blastopore ventral region. Analysis of cell shape in the ventral side of the blastopore showed that Y27632-injected embryos had weaker apical constriction compared to control and buffer-injected embryos (Figures 6L-6N). Quantification of apical constriction in transverse and sagittal sections showed an equal reduction of constriction in Y27632-injected embryos in both sections (Figures 6O and 6P). A slight expansion on the apical side of cells was also observed in the buffer-injected embryos, but this may be due to the stretching of the apical surface following buffer injection.

These results suggest that the release of ventral apical constriction causes blastopore opening; however the possibility that the increase in fluid pressure in the archenteron associated with Y27632 injection causes blastopore opening cannot be ruled out. To evaluate this possibility, Y27632 was injected into the ventral side of the blastopore and the fluid pressure in the archenteron was measured through to blastopore opening. At stage 18, embryos injected with Y27632 showed no change in fluid pressure (approximately 10 Pa) before and after blastopore opening (Figures 6Q and 6S). Similarly, buffer-injected mock embryos had no significant change in fluid pressure, although no blastopore opening occurred (Figures 6R and 6S). These results demonstrate that Y27632 attenuates the upper limit of blastopore pressure resistance to approximately 10 Pa and causes the excretion of archenteron fluid without affecting the fluid pressure. These findings support that ventral apical constriction in the blastopore regulates the pushing force.

## Discussion

### Blastopore gating mechanism involves actomyosin contraction

**Figure 7:**
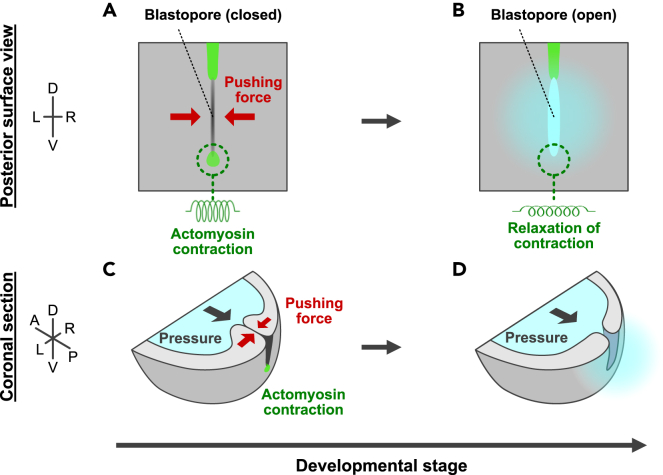
Model for blastopore gating mechanism (A-D) Model of blastopore gating mechanism. Actomyosin-dependent apical constriction at the dorsoventral end of the blastopore (light green) gives rise to a “pushing force” (red arrow) in the CBC, which maintains blastopore closure against the pressure in the archenteron (black arrow) (A, C). Relaxation of ventral contraction causes blastopore opening and fluid excretion due to a decrease in the upper limit of pressure resistance (B, D).

In this study, we show that blastopore closure is maintained in early *Xenopus* embryos by actomyosin-dependent apical constriction at the dorsoventral ends that generates a pushing force between CBCs (Figures 7A-7D). Since this pushing force can deform the CBC contact surface, the CBC tissue must be flexible. Therefore, this finding predicts that the blastopore maintains closure by bringing the flexible CBCs tightly together at both dorsoventral ends. Similar pressure valves are commonly observed in adult organisms. For example, the urethra has a flexible spongy submucosa that maintains closure through tightening entirely by the urethral sphincter.^41^^,^^42^ There is also a flexible anal cushion in the anus, which is tightened entirely from the outside by the anal sphincter.^43^^,^^44^^,^^45^ Similar structures are also observed in pressure valves for industrial products, such as air inlets for soft tennis balls.^46^ One major difference between the blastopore and pressure valves in adults is that blastopore closure relies on apical constriction, rather than a sphincter, and only the dorsoventral ends are tightened, rather than the entire blastopore. Muscles form stable structures called sarcomeres, in which actin and myosin filaments overlap. Similarly, apical constriction is driven by actomyosin, which contains actin fibers that are repeatedly polymerized and depolymerized. As such, the actomyosin structure is more dynamic than that of sarcomeres and the contraction ratio for apical constriction is higher than that for muscle contraction.^47^ The adult anus and urethra require the contraction of the entire tube by the sphincter to maintain closure, whereas the high contraction rate of apical constriction may allow the blastopore to remain closed simply by tightening the dorsoventral ends.

### Temporal regulation of fluid excretion in left-right axis formation

We found that the removal of fluid in the archenteron at the early neurula stage (stage 14) causes defects in laterality. The left-right axis is determined by the leftward flow of fluid driven by ciliary motility in the archenteron. Cilia in the gastrocoel roof plate (GRP) are tilted in the posterior direction and rotate clockwise. Thus, the rightward flow decelerates due to viscous friction and the leftward flow is dominant.^34^ There are at least two possibilities to explain why early removal of archenteron fluid disrupts left-right axis formation. The early removal may prevent the leftward flow driven by ciliary movement, or it could inhibit cilia formation or rotational movement. The formation of rotational cilia requires tension associated with convergent extension in the region that forms the GRP during the late gastrula stage.^48^ Therefore, early fluid removal may have reduced GRP tension and caused cilia dysplasia.

On the other hand, no significant effects on left-right axis formation were observed in the early excretion associated with the injection of Y27632. Since the period of fluid excretion induced by Y27632 was brief, a sufficient amount of fluid would have remained in the archenteron. Consistent with this idea, a sufficient amount of fluid excretion was observed in the collapse of the archenteron in Y27632-injected embryos. In addition, Y27632 was injected at the late neurula stage (stage 18), which may have been too late to affect ciliogenesis.

Together, our results show that fluid is retained in the archenteron until the left-right axis is determined and that the timing of fluid excretion is temporally regulated.

### Pressure measurement in embryogenesis

In this study, pressure measurements in the archenteron using microelectrodes revealed that a decrease in the upper limit of the blastopore pressure resistance causes blastopore opening. Recently, direct or indirect methods were attempted to measure pressure in the embryonic cavity to elucidate the role of fluid pressure in development.^13^ The direct method essentially detects fluid flowing into the glass needle due to pressure differences. In addition to microelectrodes,^17^ glass needles equipped with pressure sensors^19^ have been used, or pressure was measured based on the shape of the interface between the oil and body fluid inside a glass needle.^49^ To measure intracellular pressure, a nanomechanical device with Fabry-Pérot cavity or nanoparticles that exhibit fluorescence spectrum changes in response to pressure have been developed.^50^^,^^51^^,^^52^ Indirect methods were also developed to estimate the pressure in a cavity following a shape change that occurs when a force is applied to the cavity wall. Specifically, pressure is estimated by placing the embryo between gels having known stiffness^53^ or by applying force to the cavity wall with magnetic tweezers.^54^ However, these methods are affected by the wall stiffness and thus the physical properties of the cavity wall must be measured or estimated. Noninvasive estimation of pressure in the cavity from the cell shape of the cavity wall has been attempted.^55^ In this study, we used microelectrodes that allowed us to directly measure the weak fluid pressure in the archenteron. Because the microelectrode measures pressure using the difference in salt concentration between the buffer in the needle and the incoming body fluid, the salt concentration of the incoming liquid should be the same before (medium) and after (archenteron fluid) needle insertion. In particular, since the effects of salt concentration cannot be ignored in the measurement of weak pressure, in this study the salt concentration of the medium was adjusted to match that of the archenteron fluid.

Pressure measurement using microelectrodes can be improved to address the possibility that fluid could leak from the insertion site due to high fluid pressure or debris that clogs the needle tip during prolonged measurements. Further improvements in instruments to measure pressure are expected to provide a better understanding of dynamic pressure changes during embryogenesis.

### Comparison of fluid excretion mechanisms

In the mouse blastocoel cavity, fluid drains due to the partial disruption of TJs in random regions of dividing cells.^17^ Since this cellular-level excretion does not occur when the fluid pressure in the blastocoel is low, fluid pressure in the cavity would likely need to increase and exceed the upper limit of pressure resistance at the cell division site.^17^ In contrast, *Xenopus* embryos excrete from a localized tissue, the blastopore. We measured the upper limit of pressure resistance of the blastopore by combining pressurization with buffer injection and pressure measurement using microelectrodes, and found that a decrease in pressure resistance induces blastopore opening. Measurements of pushing force using a tungsten wire also supported a decrease in pressure resistance. These results indicate that the primary factor causing fluid excretion is an increase in fluid pressure in the blastocoel in mouse embryos and a decrease in the pressure resistance of the blastopore in *Xenopus* embryos, suggesting that they regulate excretion via different mechanisms. Furthermore, there is a difference between mice and *Xenopus*: random ejection at the cellular level and localized excretion at the tissue level. One possible explanation for this difference in randomness and localization may be differences in the cavity wall structure. The mouse embryo blastocoel wall has a single-layer epithelium, and thus transient rupture of epithelial TJs would allow efficient fluid drainage.^17^ In contrast, *Xenopus* embryo archenteron walls have thick tissue comprising endothelium, mesenchyme, and epithelium. Thus, fluid excretion due to epithelial collapse may be more difficult in *Xenopus* than in mice. In addition, during the late neurula stage, various tissues such as neural tube,^56^ somites,^57^ and ventral blood islands^58^ have formed, and fluid excretion from random areas may result in abnormal tissue formation. These problems could be avoided in *Xenopus* embryos through the excretion of fluid only through the blastopore. Consistent with these considerations, in fluid drainage in the inner ear of zebrafish that occurs repeatedly during the formation of the semicircular canal associated with cavity wall deformation, is mediated in a localized area, the endolymphatic sac.^18^^,^^59^ Thus, early embryos may have appropriate excretion pathways and mechanisms depending on their developmental stage and cavity structure.

### Material properties of the circumblastoporal collars

In this study, we revealed that actomyosin contraction at both dorsoventral ends of the blastopore drives the pushing force and that relief of ventral contraction induces the collapse of the archenteron. To effectively close the blastopore by the contraction of the dorsoventral ends, the CBC must have a certain degree of stiffness in order to transmit the pushing force from both ends to the center (e.g., two long narrow balloons connected at both ends). Therefore, this softening of the CBC could trigger the opening of the blastopore. We used a tungsten wire to measure the force required to open the blastopore and found that the force was reduced in later stages (Figures 4M and 4N). In addition, using this method, we revealed that the force required to stretch the CBC was comparable between stages 18 and 20 (Figures S6D-S6F). This indicates that the stiffness of the CBC is nearly constant, or that the change in stiffness is too small to detect with the tungsten wire. These results show that the primary factor in blastopore opening is release of the pushing force; however, the possibility remains that a change in CBC stiffness could contribute cooperatively to the gating mechanism. More detailed stiffness measurements using atomic force microscope (AFM) may provide a better understanding of the relationship between the material properties of CBC and the gating mechanism.

### The regulatory mechanisms of actomyosin contraction in the blastopore

Previous studies reported that actomyosin contraction is important to decrease the IMZ circumference.^27^^,^^28^ In this study, we found that actomyosin contraction at both dorsoventral ends is involved in subsequent maintenance of blastopore closure. Furthermore, we revealed that the relief of contraction at the ventral end results in fluid excretion after Y27632 injection.

Recently, various methods involving light were developed to control actomyosin contraction and relaxation. In actomyosin-dependent apical constriction, Ras homolog family member A (RhoA) localized to the apical surface of the cell is activated by Rho guanine nucleotide exchange factor (RhoGEF), after which active RhoA activates Rho kinase (ROCK). Further, active ROCK phosphorylates MLC and inhibits pMLC dephosphorylation by myosin light chain phosphatase (MLCP) to cause myosin-mediated apical constriction.^60^^,^^61^ Thus, a method has been developed to enhance contraction in a light-dependent manner by fusing exogenous domains that enhance interaction between RhoGEF and RhoA in response to specific light wavelengths.^62^^,^^63^ Light-mediated methods to relax contraction have also been reported, such as the localization of RhoGEF to the mitochondrial outer membrane to inhibit RhoA activation,^63^ or localization of Protein Phosphatase 1c to the apical surface of cells to promote myosin dephosphorylation.^64^ Spatiotemporal control of actomyosin contraction at the ventral end using these methods would allow us to elucidate the gating mechanism of the blastopore in more detail.

The molecular mechanism by which actomyosin contraction at the ventral end is resolved during the late neurula stage remains elusive. In the convergent extension movement of the neural tube, the Wnt/PCP pathway regulates actomyosin contraction as an upstream signal.^30^ In addition, the Acitivin/Nodal pathway regulates actomyosin contraction in bottle cells via RhoGEF, plekghg5.^65^ Identification of upstream signals involved in actomyosin contraction at the ventral end of the blastopore would also provide a more detailed understanding of the molecular mechanisms of blastopore gating.

In summary, this study revealed that apical constriction at the dorsal and ventral ends contributes to maintaining closure of the blastopore, and that collapse of the archenteron is caused by the relaxation of contractility at the ventral end of the blastopore. Additional features of the design principles of the blastopore remain to be characterized, and identification of upstream factors is needed. However, our results show that tissue-level opening of the blastopore and fluid excretion can be temporally controlled via actomyosin contraction even in early embryos that have immature functional muscles. As morphogenesis mediated by actomyosin contraction is universally observed in embryogenesis, we could speculate that actomyosin contraction plays an important role in controlling fluid transport in embryos of other animals as well.

### Limitations of the study

There are several limitations in the present study. First, the molecular mechanisms of blastopore opening remain to be defined. Identification of upstream signals involved in actomyosin contraction would enhance our understanding of the gating mechanism at the molecular level. Second, the spatiotemporal resolution of actomyosin contraction analysis in the blastopore needs to be further improved using optogenetics and other techniques. Since Y27632 diffuses through tissues, the possibility that Y27632 affects other tissues besides the CBC cannot be completely ruled out. Third, we cannot ignore the possibility that the excision and fixation of embryos may have had secondary effects such as tissue deformation. Time-lapse imaging deep within the live embryos using X-ray image analysis^66^ and other techniques will be important for a more detailed understanding of the gating mechanism.

## STAR★Methods

### Key resources table

**Table:** 

REAGENT or RESOURCE	SOURCE	IDENTIFIER
**Antibodies**		

Rabbit anti-Phospho-Myosin Light Chain 2 (Ser19)	Cell Signaling Technology	Cat # 3671; RRID: AB_330248
Mouse anti-E-cadherin	BD Biosciences	Cat # 610181; RRID: AB_397580
Alexa Fluor 488 goat anti-rabbit IgG (H + L)	Thermo Fisher Scientific	Cat # A11029; RRID: AB_2534088
Alexa Fluor 546 goat anti-rabbit IgG (H + L)	Thermo Fisher Scientific	Cat # A11030; RRID: AB_2534089

**Chemicals, peptides, and recombinant proteins**		

Dextran, Alexa Fluor™ 488; 10,000 MW	Thermo Fisher Scientific	Cat #D22910
Dextran, Alexa Fluor™ 568; 10,000 MW	Thermo Fisher Scientific	Cat #D22912
Dextran, Alexa Fluor™ 647; 10,000 MW	Thermo Fisher Scientific	Cat #D22914
Alexa Fluor™ 488 Hydrazide	Thermo Fisher Scientific	Cat # A10436
Phenol red solution	Sigma-Aldrich	Cat #P0290
Y27632	Nacalai Tesque	Cat # 08945
Methylhydrogen silicone fluid (KF-99)	Shin-Etsu Silicone	Cat # KF-99-1

**Experimental models: Organisms/strains**		

Wild type and albino *Xenopus laevis* frogs	Hamamatsu Seibutsu Kyozai, Watanabe Zoushoku	N/A

**Software and algorithms**		

ImageJ/Fiji (ver. 1.53t)	Schindelin et al.	RRID: SCR_002285
GraphPad Prism 8 (ver. 8.4.3)	GraphPad Software	RRID: SCR_002798
Leica Application Suite X (ver. 3.5.7)	Leica Microsystems	RRID: SCR_013673
Labchart 7	ADInstruments	RRID: SCR_001620

**Other**		

Aluminum foil (d: 5 μm)	Nilaco	Cat # AL-013131
Tungsten wire (φ: 30 μm)	Nilaco	Cat # W-461097
950Ag wire (φ: 0.4 mm)	Comokin	N/A
Glass capillary	Narishige	Cat # GC-1
FluoSpheres™ Size Kit #2, Carboxylate-modified Microspheres, yellow-green fluorescent (505/515), 2% solids, six sizes	Thermo Fisher Scientific	Cat #F8888

### Resource availability

#### Lead contact

Further information and requests for resources and reagents should be directed to and will be fulfilled by the lead contact, Soichiro Kato (soichiro.kato@riken.jp).

#### Materials availability

This study did not generate new unique reagents.

### Experimental model and subject details

#### X. laevis

Adult *Xenopus laevis* were purchased from domestic breeders (Hamamatsu Seibutsu Kyozai, Shizuoka, Japan; Watanabe Zoshoku, Hyogo, Japan). Ovulation was induced by injecting mature females with 600 units of human chorionic gonadotropin (Gonatropin 3000; ASKA Animal Health, Tokyo, Japan). Spawned eggs were fertilized *in vitro* by mixing with sperm suspended in 1.3x Barth’s medium (13 mM HEPES-NaOH/pH 7.4, 114.4 mM NaCl, 1.3 mM KCl, 0.533 mM CaCl_2_, 0.429 mM Ca(NO_3_)_2_, 1.066 mM MgSO_4_, 3.12 mM NaHCO_3_).

### Method details

#### Time-lapse imaging of extracellular fluid movement and imaging analysis

To label embryonic cells, 10 kDa Alexa Fluor 488-dextran (Alexa 488-dex) (D22910; Thermo Fisher Scientific, Massachusetts, USA) or 10 kDa Alexa Fluor 647-dextran (Alexa 647-dex) (D22914; Thermo Fisher Scientific) (total 10–20 ng) was injected into the two blastomeres at the 2-cell stage. At stage 14, archenteron fluid was visualized by injecting 10 kDa Alexa Fluor 568-dextran (Alexa 568-dex) (D22912; Thermo Fisher Scientific) (2 nL, 10 mg/mL). Time-lapse images were acquired using an MVX-10 stereomicroscope (Olympus, Tokyo, Japan) equipped with an EM-CCD camera (ImagEM; Hamamatsu Photonics K.K., Hamamatsu, Japan), a BZ-X710 fluorescence microscope (Keyence, Osaka, Japan) with a 2× objective (Plan-Apo 2×/0.1; Nikon, Tokyo, Japan), an IX83 microscope (Olympus) equipped with a spinning disk unit (CSU-W1; Yokogawa, Tokyo, Japan), or an EM-CCD camera (iXon Ultra 888; Andor, Belfast, UK), and 20× objective (UPlanSApo 20×/0.75 DRY; Olympus). Fluorescence images for fixed embryos were acquired using an SP8 confocal microscope (Leica Microsystems GmbH, Wetzlar, Germany) with a 10× (HC PL APO CS2 10×/0.40 DRY; Leica), 20× (HC PL APO CS2 20×/0.75 DRY; Leica), or 40× (HC PL APO CS2 40×/1.30 OIL; Leica) objective.

#### Analysis of fluid excretion timing

To examine fluid excretion, Alexa 568-dex was injected into the archenteron of embryos with (VM+) or without the vitelline membrane (VM-) at stage 13–14. The vitelline membrane was removed with forceps at stage 14. Time-lapse images were acquired using an MVX-10 stereomicroscope (Olympus) equipped with an EM-CCD camera (ImagEM; Hamamatsu Photonics K.K.). Embryos without the vitelline membrane that had excreted fluid were immediately fixed in trichloroacetic acid solution (TCA) containing 1.85% formaldehyde, after which the developmental stage was analyzed. For embryos with the vitelline membrane, the membrane was removed and the embryos were cultured at 12 °C for 10 min prior to fixation.

#### Early removal of archenteron fluid

For early removal of archenteron fluid, the anteroventral archenteron wall was perforated with a glass needle and the hole was maintained for 30 min by repeated insertion of the needle. Although most of the archenteron fluid was excreted, the ventral side of the embryos was gently pushed to ensure complete removal of the fluid. Embryos were cultured until stage 45 and laterality was analyzed by the direction of heart tube and gut coiling.

#### Imaging of beads excretion by perforation

To examine archenteron pressure, 1 μm fluorescent beads (20 nL, FluoSpheres carboxylate-modified microspheres with yellow-green fluorescence; Thermo Fisher Scientific) diluted in LCMR (1/200) supplemented with 1% skim-milk (232100; BD, New Jersey, USA) were injected into the archenteron at stage 18. Then, the archenteron wall was perforated with a glass needle and movies were acquired using an MVX-10 stereomicroscope (Olympus) equipped with an EM-CCD camera (ImagEM; Hamamatsu Photonics K.K.).

#### Fluid pressure measurement

To measure archenteron hydrostatic pressure, analog signals from a Micropressure system (900A; WPI, Sarasota, USA) were converted to digital signals with an AD converter (PL2602; ADInstruments, Dunedin, New Zealand) and the data was then analyzed with Labchart software (Labchart 7; ADInstruments, Dunedin, New Zealand). Microelectrodes were made by stretching a glass capillary (GC-1; inner diameter = 0.6 mm) with a needle puller (PC-10) and adjusting the tip inner diameter to 7.4 ± 0.8 μm. If the tip diameter is too large, buffer will leak from the needle, and needles having too small tip diameters can become clogged with cellular debris that compromises pressure measurements. The probe tip was coated with silicon (Sigmacote; Sigma-Aldrich, St. Louis, USA) prior to measurement. A probe filled with 3 M NaCl solution equipped with a dial gauge (73750; Shinwa, Niigata, Japan) was connected to a micromanipulator (MN-153; Narishige). Probes were calibrated based on changes in hydrostatic pressure by submerging the needle tip to depths of 0 mm and 20 mm. Changes in water depth during pressure measurement were corrected using a calibration curve of the dial gauge value and water pressure changes.

To examine electrical resistance at stage 18 or 20–21, archenteron fluid was collected from 10 embryos. After debris was removed by centrifugation (4 °C, 15300 × *g*, 1 min), the collected supernatant was diluted 10-fold and used to fill a glass capillary (GC-1; 90 mm length, 0.6 mm inner diameter). Ag wires (Comokin, Tokyo, Japan; 0.4 mm diameter) coated with AgCl were inserted into the capillary and electrical resistance of fluid in the capillary was measured using a digital multimeter (3244-60; Hioki, Nagano, Japan) (St. 18, n = 7; St. 20, n = 5). To create an Arc-buffer having the same electrical resistance as the archenteron, 0.1x Barth’s medium containing various concentrations of NaCl (8.8–100 mM) was diluted 10-fold and the electrical resistance was measured using the same method (n = 5). A calibration curve generated from 0.1x Barth’s medium containing various concentrations of NaCl as a function of electrical resistance was used to determine the Arc-buffer composition. To measure the upper limit of pressure resistance, Arc-PR-buffer (10% phenol red solution (P0290; Sigma Aldrich) in Arc-buffer), was injected into the archenteron. When water pressure at the same depth was measured with a microelectrode using Arc-buffer or archenteron fluid from stage 18 or 20–21, no significant differences were observed.

To examine the effects of Arc-buffer on fluid excretion, embryos injected with 10 kDa Alexa 568-dex (2 nL, 10 mg/mL) into the archenteron were cultured in 0.1x Barth’s medium or Arc-buffer. There was no difference in the timing of fluid excretion for either buffer.

To measure archenteron pressure, embryos from which the vitelline membrane was removed at stage 14 were injected with 10 kDa Alexa 568-dex (2 nL, 10 mg/mL) into the archenteron and then placed laterally (measurement of pressure and pressure resistance) or posteriorly (measurement of pressure after injection of Y27632) in the hollow of an agarose plate filled with Arc-buffer.

To measure blastopore pressure resistance, the pressure probe was first inserted into the archenteron to measure fluid pressure. The needle for injecting buffer was then inserted and Arc-PR-buffer was injected continuously until fluid leaked from the blastopore. The pressure in the archenteron at which fluid leaked out was defined as the upper limit of pressure resistance of the blastopore. Buffer was injected into the archenteron at three different injection rates (5 nL/s, 2.5 nL/s, 1.25 nL/s) and no significant difference in Pres was observed among the different rates. Time-lapse images were acquired using an SZX-7 stereomicroscope (Olympus) equipped with a digital camera (ILCE-QX1; Sony, Tokyo, Japan).

#### Transmission electron microscopy (TEM) analysis

Embryos were fixed in a solution of 2.5% glutaraldehyde and 2% paraformaldehyde in 50 mM HEPES buffer, pH7.4, at 4 °C overnight. After primary fixation, the samples were washed three times with 50 mM HEPES buffer (pH 7.4) for 10 min and postfixed with 2% osmium tetroxide solution for 3 h at 4 °C. Then, the samples were washed three times with 50 mM HEPES buffer (pH7.4) and stained with 0.25% aqueous uranyl acetate for 2 h at room temperature. After staining, the samples were washed three times with distilled water for 10 min at room temperature, dehydrated with a graded ethanol series (30%, 50%, 70%, 90%, 100%) for 15 min at room temperature, bathed in propylene oxide for 15 min at room temperature, and embedded in epoxy resin (EPON812; Nisshin-EM, Tokyo, Japan) for 48 h at 60 °C. Ultrathin sections were stained with 2% uranyl acetate followed by lead stain solution and analyzed by TEM (H-7600; Hitachi, 100 kV).

#### Insertion of aluminum foil into the blastopore

To visualize embryonic cells, 10 kDa Alexa 488-dex (total 10 ng) was injected into two blastomeres at the 2-cell stage. At stage 13–14, archenteron fluid was labeled by injecting 10 kDa Alexa 568-dex (2 nL, 10  mg/mL) into the archenteron. A 5 μm-thick, ∼100 μm-wide strip of aluminum foil (AL-013131; Nilaco, Tokyo, Japan) coated with 0.1% BSA was inserted into the blastopore of stage 14 embryos with the vitelline membrane removed. To confirm proper insertion, embryos were fixed with 2% TCA and 9.25% formaldehyde and then cleared with benzyl alcohol/benzyl benzoate (BABB) solution. Fluorescence images were acquired using an SP8 confocal microscope with a 10× objective (Leica).

Time-lapse images were acquired using a BZ-X710 fluorescence microscope (Keyence) with a 2× objective (Nikon). To prevent flow of culture medium, high-viscosity 0.1x Barth’s medium containing 3% methyl cellulose (#1500; Nacalai Tesque, Kyoto, Japan) was used.

#### Insertion of a glass bead into the blastopore

A glass bead (approximately 130 μm diameter) made by heating the tip of a glass needle with a gas torch until a bead formed was inserted into the blastopore. Time-lapse images were acquired using an SZX-16 stereomicroscope (Olympus) equipped with a digital camera (DP80; Olympus). Blastopore closure after removing the glass bead was analyzed in ImageJ using the shading of the hole as an indicator.

#### Release of pushing force by excision

To visualize embryonic cells, 10 kDa Alexa 488-dex (total 10 ng) was injected into two blastomeres at the 2-cell stage. To release the CBC pushing force, the tissue around the blastopore was excised, leaving the dorsal side intact. Then, the ventral side of the blastopore slit was cut and embryos were fixed immediately (within 1 min) in 2% TCA and 1.85% formaldehyde. Fixed embryos were dehydrated in methanol and cleared with BABB solution. Fluorescence images were acquired using an SP8 confocal microscope with a 10× objective (Leica).

To examine the CBC shape without incision, 2% TCA and 1.85% formaldehyde were continuously injected into the archenteron at the excretion stage. Fixed and cleared embryos were analyzed using an SP8 confocal microscope with a 10× objective (Leica).

#### Geometrical analysis of the blastopore

To analyze the shape of the CBC with the ventral end of the blastopore resected, an optical coronal section corresponding to the center of the blastopore slit was obtained from embryos cleared with BABB. The fluorescence images were then binarized using ImageJ to obtain the outline of the CBC. For control embryos and embryos having ventral ends that were not cut, the outline was obtained manually. The direction perpendicular and parallel to the slit was set as the x axis and y axis, respectively. The length with the longest y axis distance was defined as the thickness of the CBC (Figure S5F).

For curvature analysis, the point on the outline that fell midpoint between the maximum and minimum values of the y axis was set to A. Further, points on the outline ±32 μm away from point A in the y axis direction were set as B (+32 μm) and C (- 32 μm). Curvature was calculated according to the following equation:

$$curvature=\frac{2h}{{\left(\frac{w}{2}\right)}^{2}+h^{2}}$$

*h* and *y* were obtained from the following equation.

$$h=\left|A_{x}-\frac{B_{x}-C_{x}}{2}\right|$$

$$w=\left|B_{y}-C_{y}\right|$$

Here, the subscripts *x* and *y* indicate the x and y coordinates of the respective points.

The length of the contact surface of the slit was defined as the length of a straight line connecting the endpoints of the contact surface determined using ImageJ.

#### Force and stiffness measurement using a tungsten wire

To make a force measurement probe, a tungsten wire (30 μm diameter; W-461097; Nilaco) was inserted into the tip of a bent glass needle and fixed with UV resin (BD-SKEJ; Bondic, Pennsylvania, USA). To calibrate the force measurement probe, the tungsten wire was placed horizontally and 20 nL silicon oil (KF-99; Shin-Etsu Silicone, Tokyo, Japan) was repeatedly added to the tip of the wire up to a volume of 400 nL. The probe tip displacement was obtained using an SZX-7 stereomicroscope (Olympus; tilted 90°) equipped with a digital camera (ILCE-QX1; Sony) and the values were used to make a force-displacement curve.

To measure the force required to open the blastopore, embryos with archenteron fluid labeled with phenol red solution were placed in the hollow of an agarose plate with the posterior side facing up. The pulling probe and measurement probe were inserted separately on both sides of the slit, and only the pulling probe was moved horizontally until the blastopore opened. The opening of the blastopore was detected by leakage of archenteron fluid labeled with phenol red. Time-lapse images were acquired using an SZX-16 stereomicroscope (Olympus) equipped with a digital camera (DP80; Olympus) and the force required to open the blastopore was calculated.

To measure CBC stiffness, embryos were placed in the hollow of an agarose plate with the posterior side facing up. The pulling and measurement probes were both inserted into one side of the slit, and only the pulling probe was moved to stretch the CBC in the direction of the slit long axis. Time-lapse images were acquired using an SZX-7 stereomicroscope (Olympus) equipped with a digital camera (ILCE-QX1; Sony) and the force required to stretch the CBC was calculated.

#### Immunofluorescence staining

To visualize cells, 10 kDa Alexa 647-dex (total 10 ng) was injected into the two blastomeres at the 2-cell stage. At stage 14, 10 kDa Alexa 568-dex (2 nL, 10 mg/mL) was injected into the archenteron, after which the vitelline membrane was removed. Embryos were then fixed at the desired stage in 2% TCA for 20 min followed by washing three times in PBS-T buffer (0.3% polyoxyethylene (10) octylphenyl ether in PBS). Thereafter, the peripheral tissue of the blastopore was excised and blocked with blocking buffer (PBS-T buffer containing 10% FBS). Fragmented tissues were then incubated overnight at 4 °C with anti-phosphorylated myosin light chain (1:100 dilution, #3671, CST, Massachusetts, USA) and anti-E-cadherin (1:200 dilution, 610181, BD Bioscience, New Jersey, USA) as primary antibodies that were diluted in Can Get Signal B solution (Toyobo, Osaka, Japan). Tissues were then washed with PBS-T (3 × 30 min) and incubated with Alexa Fluor 488 anti-rabbit IgG (1:200 dilution, A-11029, Thermo Fisher Scientific) or Alexa Fluor 546 anti-mouse IgG (1:500 dilution, A-11030, Thermo Fisher Scientific) as secondary antibodies diluted in Can Get Signal B solution. After washing tissues with PBS-T buffer, tissues were dehydrated in methanol and cleared with BABB solution. Fluorescent images were acquired using an SP8 confocal microscope with a 40× objective (Leica).

#### Apical constriction in the ventral end of the blastopore

To analyze apical constriction in the mediolateral direction, serial optical transverse sections were acquired using an SP8 confocal microscope with a 40× objective (Leica). To analyze apical constriction in the anteroposterior direction, sagittal sections were reconstructed using serial optical transverse sections. The apical width and apical-basal length of cells in the ventral end of the blastopore were measured and the ratio between them was calculated.

#### Inhibition of actomyosin contraction using Y27632

For time-lapse imaging of embryos injected with Y27632, 10 kDa Alexa 647-dex was injected into two blastomeres at the 2-cell stage to label embryonic cells. At stage 14, 10 kDa Alexa 568-dex was injected into the archenteron, and the vitelline membrane was removed. At stage 18, 1 nL Y27632 solution (LCMR containing Y27632 (08945; Nacalai Tesque; 0.64 ng) and Alexa Fluor 488 hydrazide (12.5 ng; A10436; Thermo Fisher Scientific)) or Mock solution (LCMR containing 12.5 ng Alexa Fluor 488 hydrazide) was injected into the CBC tissue at the blastopore ventral region. Time-lapse images were acquired 1 min after injection of Y27632 or Mock solution using an MVX-10 stereomicroscope (Olympus) equipped with an EM-CCD camera (ImagEM; Hamamatsu Photonics K.K.). Embryos that excreted archenteron fluid within 10 min of injection were counted and further cultured until stage 20 to assess developmental abnormalities.

To examine the effect of Y27632 injection on myosin phosphorylation, 10 kDa Alexa 647-dex was injected into two blastomeres at the 2-cell stage. After injection of Y27632 solution or Mock solution without Alexa Fluor 488 hydrazide, embryos were fixed and used for immunofluorescence staining with anti-pMLC antibody.

To assess the effect of Y27632 injection on archenteron hydrostatic pressure, archenteron fluid was first labeled with 10 nL phenol red solution. Embryos were then set in the hollow of agarose plate with the blastopore facing upward. Y27632 or mock solution was injected while measuring pressure. Time-lapse images were acquired using an SZX-7 stereomicroscope equipped with a digital camera (ILCE-QX1). Blastopore opening was detected by excretion of phenol red-labeled fluid.

### Quantification and statistical analysis

No statistical methods were used to predetermine sample size. Each experiment was performed at least three times. All statistical analyses were performed using GraphPad Prism 8 software (GraphPad Software, San Diego, CA, USA). All data were evaluated for normal distribution and equal distribution by normality test and F-test, respectively. For parametric data, an unpaired t test (Figures 4K, 4L, and S6F) or ordinary one-way ANOVA (Figures S2D and S4D) was used to evaluate differences between two groups. For non-parametric data, Mann-Whitney U test (Figures 2J and 4N) or Kruskal-Wallis test (Figures 1J, 2E, 4J, 5T, 5U, 6O, 6P, S2C, and S7G) was used to evaluate differences between two groups. Differences in the laterality defect rate were evaluated using Fischer’s exact test (Figure 1P).

## Data Availability

• All data reported in this paper will be shared by the lead contact upon request. • This paper does not report original code. • Any additional information required to reanalyze the data reported in this paper is available from the lead contact upon request.

## References

[1] Hall J.E. (2015).

[2] Spring K.R. (1999). Epithelial fluid transport—a century of investigation. News Physiol. Sci..

[3] Fischbarg J. (2010). Fluid transport across leaky epithelia: central role of the tight junction and supporting role of aquaporins. Physiol. Rev..

[4] Rosenthal R., Milatz S., Krug S.M., Oelrich B., Schulzke J.D., Amasheh S., Günzel D., Fromm M. (2010). Claudin-2, a component of the tight junction, forms a paracellular water channel. J. Cell Sci..

[5] Rosenthal R., Günzel D., Piontek J., Krug S.M., Ayala-Torres C., Hempel C., Theune D., Fromm M. (2020). Claudin-15 forms a water channel through the tight junction with distinct function compared to claudin-2. Acta Physiol..

[6] Ayala-Torres C., Krug S.M., Schulzke J.D., Rosenthal R., Fromm M. (2019). Tricellulin effect on paracellular water transport. Int. J. Mol. Sci..

[7] Agre P., Preston G.M., Smith B.L., Jung J.S., Raina S., Moon C., Guggino W.B., Nielsen S. (1993). Aquaporin CHIP: the archetypal molecular water channel. Am. J. Physiol..

[8] Day R.E., Kitchen P., Owen D.S., Bland C., Marshall L., Conner A.C., Bill R.M., Conner M.T. (2014). Human aquaporins: regulators of transcellular water flow. Biochim. Biophys. Acta.

[9] Ku D.N. (1997). Blood flow in arteries. Annu. Rev. Fluid Mech..

[10] Kawabe Y., Mizobe K., Bando Y., Sakiyama K., Taira F., Tomomura A., Araki H., Amano O. (2016). Morphological changes of myoepithelial cells in the rat submandibular gland following the application of surgical stimuli. Acta Histochem. Cytochem..

[11] Andersson K.E., Arner A. (2004). Urinary bladder contraction and relaxation: physiology and pathophysiology. Physiol. Rev..

[12] Jung J., Ahn H.K., Huh Y. (2012). Clinical and functional anatomy of the urethral sphincter. Int. Neurourol. J..

[13] Chan C.J., Hiiragi T. (2020). Integration of luminal pressure and signalling in tissue self-organization. Development.

[14] Chugh M., Munjal A., Megason S.G. (2022). Hydrostatic pressure as a driver of cell and tissue morphogenesis. Semin. Cell Dev. Biol..

[15] Schliffka M.F., Tortorelli A.F., Ozguc O., de Plater L., Polzer O., Pelzer D., Maitre J.L. (2021). Multiscale analysis of single and double Maternal-Zygotic Myh9 and Myh10 mutants during mouse preimplantation development. Elife.

[16] Dumortier J.G., Le Verge-Serandour M., Tortorelli A.F., Mielke A., de Plater L., Turlier H., Maître J.L. (2019). Hydraulic fracturing and active coarsening position the lumen of the mouse blastocyst. Science.

[17] Chan C.J., Costanzo M., Ruiz-Herrero T., Mönke G., Petrie R.J., Bergert M., Diz-Muñoz A., Mahadevan L., Hiiragi T. (2019). Hydraulic control of mammalian embryo size and cell fate. Nature.

[18] Swinburne I.A., Mosaliganti K.R., Upadhyayula S., Liu T.L., Hildebrand D.G.C., Tsai T.Y.C., Chen A., Al-Obeidi E., Fass A.K., Malhotra S. (2018). Lamellar projections in the endolymphatic sac act as a relief valve to regulate inner ear pressure. Elife.

[19] Mosaliganti K.R., Swinburne I.A., Chan C.U., Obholzer N.D., Green A.A., Tanksale S., Mahadevan L., Megason S.G. (2019). Size control of the inner ear via hydraulic feedback. Elife.

[20] Muntz L. (1975). Myogenesis in the trunk and leg during development of the tadpole of Xenopus laevis (Daudin 1802). J. Embryol. Exp. Morphol..

[21] Chanoine C., Hardy S. (2003). Xenopus muscle development: from primary to secondary myogenesis. Dev. Dyn..

[22] Brown M.G. (1941). Collapse of the archenteron in embryos of Amblystoma and Rana. J. Exp. Zool..

[23] Tuft P.H. (1962). The uptake and distribution of water in the embryo of Xenopus laevis (Daudin). J. Exp. Biol..

[24] Keller R., Shook D. (2008). Dynamic determinations: patterning the cell behaviours that close the amphibian blastopore. Philos. Trans. R. Soc. Lond. B Biol. Sci..

[25] Shook D.R., Kasprowicz E.M., Davidson L.A., Keller R. (2018). Large, long range tensile forces drive convergence during Xenopus blastopore closure and body axis elongation. Elife.

[26] Shook D.R., Wen J.W.H., Rolo A., O'Hanlon M., Francica B., Dobbins D., Skoglund P., DeSimone D.W., Winklbauer R., Keller R.E. (2022). Characterization of convergent thickening, a major convergence force producing morphogenic movement in amphibians. Elife.

[27] Skoglund P., Rolo A., Chen X., Gumbiner B.M., Keller R. (2008). Convergence and extension at gastrulation require a myosin IIB-dependent cortical actin network. Development.

[28] Pfister K., Shook D.R., Chang C., Keller R., Skoglund P. (2016). Molecular model for force production and transmission during vertebrate gastrulation. Development.

[29] Wallingford J.B., Rowning B.A., Vogeli K.M., Rothbächer U., Fraser S.E., Harland R.M. (2000). Dishevelled controls cell polarity during Xenopus gastrulation. Nature.

[30] Ewald A.J., Peyrot S.M., Tyszka J.M., Fraser S.E., Wallingford J.B. (2004). Regional requirements for Dishevelled signaling during Xenopus gastrulation: separable effects on blastopore closure, mesendoderm internalization and archenteron formation. Development.

[31] Feroze R., Shawky J.H., von Dassow M., Davidson L.A. (2015). Mechanics of blastopore closure during amphibian gastrulation. Dev. Biol..

[32] Gont L.K., Steinbeisser H., Blumberg B., Derobertis E.M. (1993). Tail Formation as a continuation of gastrulation - the multiple cell-populations of the Xenopus tailbud derive from the late blastopore lip. Development.

[33] Fainsod A., Steinbeisser H., De Robertis E.M. (1994). On the function of BMP-4 in patterning the marginal zone of the Xenopus embryo. EMBO J..

[34] Schweickert A., Weber T., Beyer T., Vick P., Bogusch S., Feistel K., Blum M. (2007). Cilia-driven leftward flow determines laterality in Xenopus. Curr. Biol..

[35] Luu O., Damm E.W., Parent S.E., Barua D., Smith T.H.L., Wen J.W.H., Lepage S.E., Nagel M., Ibrahim-Gawel H., Huang Y. (2015). PAPC mediates self/non-self-distinction during Snail1-dependent tissue separation. J. Cell Biol..

[36] Martin A.C., Goldstein B. (2014). Apical constriction: themes and variations on a cellular mechanism driving morphogenesis. Development.

[37] Heer N.C., Martin A.C. (2017). Tension, contraction and tissue morphogenesis. Development.

[38] Lee J.Y., Harland R.M. (2007). Actomyosin contractility and microtubules drive apical constriction in Xenopus bottle cells. Dev. Biol..

[39] Suzuki M., Morita H., Ueno N. (2012). Molecular mechanisms of cell shape changes that contribute to vertebrate neural tube closure. Dev. Growth Differ..

[40] Ishizaki T., Uehata M., Tamechika I., Keel J., Nonomura K., Maekawa M., Narumiya S. (2000). Pharmacological properties of Y-27632, a specific inhibitor of rho-associated kinases. Mol. Pharmacol..

[41] Suzuki Bellucci C.H., Wöllner J., Gregorini F., Birnböck D., Kozomara M., Mehnert U., Kessler T.M. (2012). External urethral sphincter pressure measurement: an accurate method for the diagnosis of detrusor external sphincter dyssynergia?. PLoS One.

[42] Pipitone F., Sadeghi Z., DeLancey J.O.L. (2021). Urethral function and failure: a review of current knowledge of urethral closure mechanisms, how they vary, and how they are affected by life events. Neurourol. Urodyn..

[43] Gibbons C.P., Trowbridge E.A., Bannister J.J., Read N.W. (1986). Role of anal cushions in maintaining continence. Lancet.

[44] Lestar B., Penninckx F., Kerremans R. (1989). The composition of anal basal pressure. An in vivo and in vitro study in man. Int. J. Colorectal Dis..

[45] Margetis N. (2019). Pathophysiology of internal hemorrhoids. Ann. Gastroenterol..

[46] Takahashi K., Toyota T., Yamaguchi D., Maki T., Maki K., Komine S., Kataoka J., Park S.J. (2017). Durability of soft-tennis ball. - comparison of 3 companies -. The Gakusen contemporary management review.

[47] Coravos J.S., Martin A.C. (2016). Apical sarcomere-like actomyosin contracts nonmuscle Drosophila epithelial cells. Dev. Cell.

[48] Chien Y.H., Srinivasan S., Keller R., Kintner C. (2018). Mechanical strain determines cilia length, motility, and planar position in the left-right organizer. Dev. Cell.

[49] Yang J., Duan X., Fraser A.K., Choudhury M.I., Ewald A.J., Li R., Sun S.X. (2019). Microscale pressure measurements based on an immiscible fluid/fluid interface. Sci. Rep..

[50] Gómez-Martínez R., Hernández-Pinto A.M., Duch M., Vázquez P., Zinoviev K., de la Rosa E.J., Esteve J., Suárez T., Plaza J.A. (2013). Silicon chips detect intracellular pressure changes in living cells. Nat. Nanotechnol..

[51] Lay A., Wang D.S., Wisser M.D., Mehlenbacher R.D., Lin Y., Goodman M.B., Mao W.L., Dionne J.A. (2017). Upconverting nanoparticles as optical sensors of nano- to micro-Newton forces. Nano Lett..

[52] Lay A., Sheppard O.H., Siefe C., McLellan C.A., Mehlenbacher R.D., Fischer S., Goodman M.B., Dionne J.A. (2019). Optically robust and biocompatible mechanosensitive upconverting nanoparticles. ACS Cent. Sci..

[53] Leonavicius K., Royer C., Preece C., Davies B., Biggins J.S., Srinivas S. (2018). Mechanics of mouse blastocyst hatching revealed by a hydrogel-based microdeformation assay. Proc. Natl. Acad. Sci. USA.

[54] Wang X., Zhang Z., Tao H., Liu J., Hopyan S., Sun Y. (2018). Characterizing inner pressure and stiffness of trophoblast and inner cell mass of blastocysts. Biophys. J..

[55] Barua D., Parent S.E., Winklbauer R. (2017). Mechanics of fluid-filled interstitial gaps. II. Gap characteristics in Xenopus embryonic ectoderm. Biophys. J..

[56] Davidson L.A., Keller R.E. (1999). Neural tube closure in Xenopus laevis involves medial migration, directed protrusive activity, cell intercalation and convergent extension. Development.

[57] Hamilton L. (1969). The formation of somites in Xenopus. J. Embryol. Exp. Morphol..

[58] Walmsley M., Ciau-Uitz A., Patient R. (2002). Adult and embryonic blood and endothelium derive from distinct precursor populations which are differentially programmed by BMP in Xenopus. Development.

[59] Munjal A., Hannezo E., Tsai T.Y.C., Mitchison T.J., Megason S.G. (2021). Extracellular hyaluronate pressure shaped by cellular tethers drives tissue morphogenesis. Cell.

[60] Mulinari S., Häcker U. (2010). Rho-guanine nucleotide exchange factors during development: force is nothing without control. Small GTPases.

[61] Denk-Lobnig M., Martin A.C. (2019). Modular regulation of Rho family GTPases in development. Small GTPases.

[62] Oakes P.W., Wagner E., Brand C.A., Probst D., Linke M., Schwarz U.S., Glotzer M., Gardel M.L. (2017). Optogenetic control of RhoA reveals zyxin-mediated elasticity of stress fibres. Nat. Commun..

[63] Valon L., Marín-Llauradó A., Wyatt T., Charras G., Trepat X. (2017). Optogenetic control of cellular forces and mechanotransduction. Nat. Commun..

[64] Yamamoto K., Miura H., Ishida M., Mii Y., Kinoshita N., Takada S., Ueno N., Sawai S., Kondo Y., Aoki K. (2021). Optogenetic relaxation of actomyosin contractility uncovers mechanistic roles of cortical tension during cytokinesis. Nat. Commun..

[65] Popov I.K., Ray H.J., Skoglund P., Keller R., Chang C. (2018). The RhoGEF protein Plekhg5 regulates apical constriction of bottle cells during gastrulation. Development.

[66] Moosmann J., Ershov A., Altapova V., Baumbach T., Prasad M.S., LaBonne C., Xiao X., Kashef J., Hofmann R. (2013). X-ray phase-contrast in vivo microtomography probes new aspects of Xenopus gastrulation. Nature.

