# Supplementary material for: Blastopore gating mechanism to regulate extracellular fluid excretion
Source: iScience. 2023 Apr 8;26(5):106585. doi: 10.1016/j.isci.2023.106585 (PMC10182286; doi:10.1016/j.isci.2023.106585)
Supplement: Document S1. Figures S1-S7 [file mmc1.pdf]

iScience, Volume *26*

Supplemental information

Blastopore gating mechanism to regulate extracellular fluid excretion

Soichiro Kato and Hidehiko Inomata

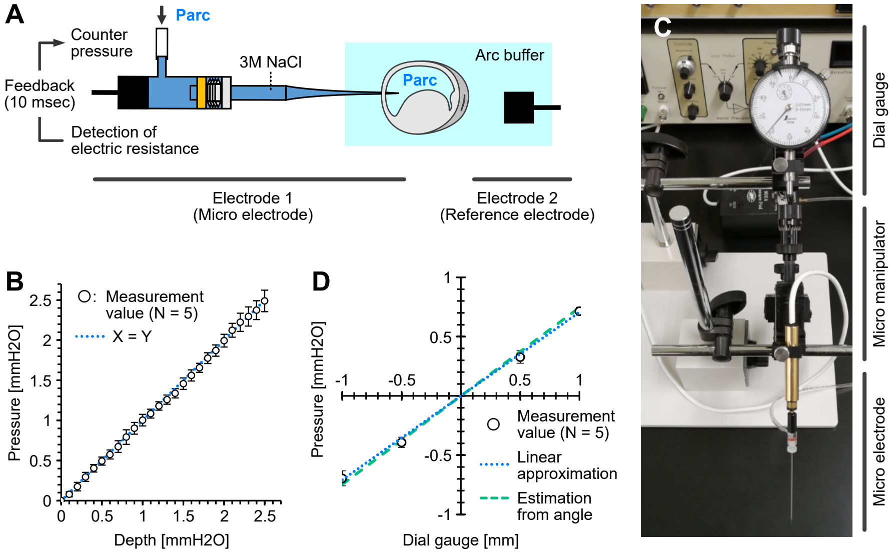

Figure S1. Measurement of fluid pressure *in vivo*, related to Figure 2.

(A) Schematic of pressure measurement using a microelectrode. Parc, fluid pressure in the archenteron.
(B) Calibration of the pressure probe with hydrostatic pressure by increasing the needle tip in 0.1 mm intervals. Error bars, ± SD (N = 5).
(C) Setting of pressure probe. A microelectrode and a dial gauge for measuring water depth were attached to the micromanipulator.
(D) Correction of the effect of depth changes on pressure measurement. Depth changes of the tip of the probe during measurement were calibrated using the dial gauge value for each experiment. Error bars, ± SD (N = 5); Blue dotted line, linear approximation of measurement data; Green dotted line, depth change calculated from the dial gauge value and angle of the probe relative to horizontal.

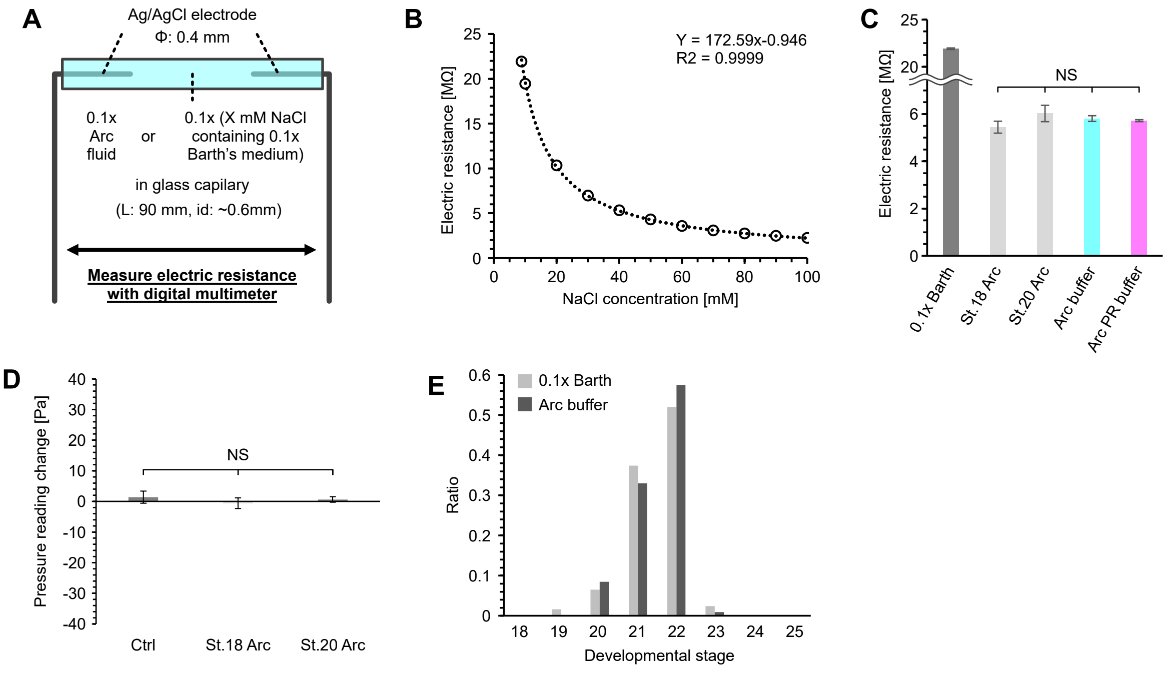

Figure S2. Effects of salt concentration in the medium on pressure measurements, related to Figure 2.

(A) Measurement of electrical resistance of archenteron fluid and buffer. Electrical resistance in a glass capillary filled with 10-fold diluted archenteron fluid or buffer was measured with a digital multimeter.
(B) Electrical resistance of buffer having varying salt concentrations (N = 5). Dotted line, exponential function fitted to measurement data; error bars, ± SD.
(C) Electrical resistance of 0.1x Barth's medium (N = 5), Arc-buffer (0.1x Barth's medium containing 36.7 mm NaCl) (N = 5), Arc-PR-buffer (0.1x Barth's medium containing 36.7 mm NaCl and phenol red) (N = 5), St.18 Arc (archenteron fluid at stage 18) (N = 7) and St.20 Arc (archenteron fluid at stage 20) (N = 7). The electrical resistance of Arc-buffer and Arc-PR-buffer is approximately equal to that of the archenteron fluid. Error bars, ± SD.
(D) Change in the pressure measurement value upon switching the medium from Arc-buffer to Arc-buffer having the same electrical resistance as the archenteron fluid at stage 18 (St.18 arc; N = 10) and stage 20 (St.20 arc; N = 9) without depth change, compared to measurements taken when the medium was not changed (Ctrl; N = 9). Error bars, ±SD.
(E) Measurement of fluid excretion stages in 0.1x Barth's medium (n = 123 embryos) and Arc-buffer (n = 106 embryos).

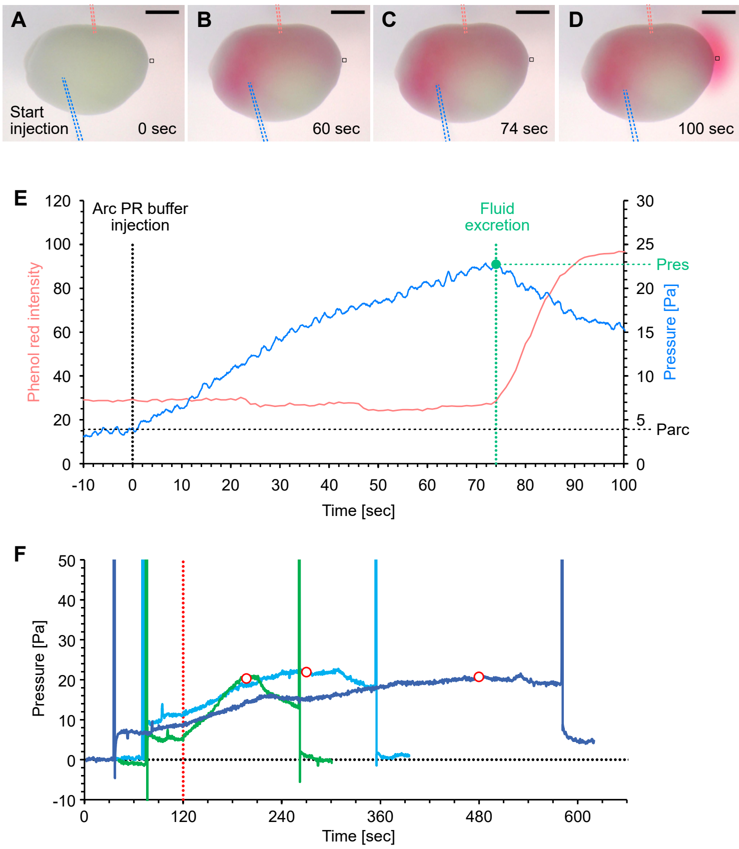

Figure S3. Measurement of the upper limit of pressure resistance in the blastopore, related to Figure 2.

(A-D) Snapshot images from representative time-lapse imaging. Arc-PR-buffer containing phenol red was injected into the archenteron from t = 0, and fluid was excreted through the blastopore at t = 74. Blue and red dotted lines represent the needle for measuring pressure and injecting Arc-PR-buffer, respectively. Scale bars = 500 µm.

(E) Pressure measurement value in (A-D). Phenol red intensity in the black circle of (A-D) is represented by a red line. The pressure at which the fluid drained was set as the upper limit of pressure resistance (Pres).

(F) Representative data for the measurement of Pres under three different injection rates. Continuous injection was started at 120 sec. Green, 5 nL/sec; blue, 2.5 nL/sec; navy, 1.25 nL/sec, red circle, blastopore opening.

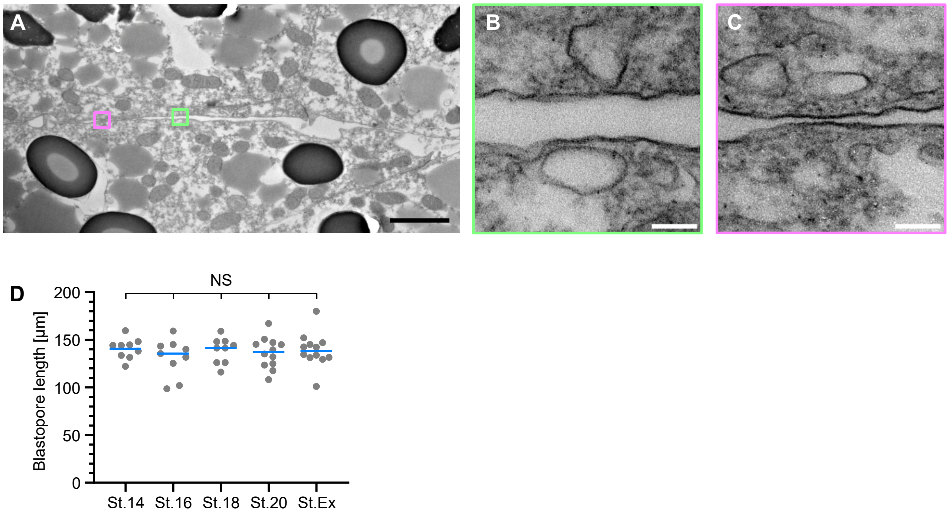

Figure S4. Transmission electron microscopy (TEM) analysis of the blastopore, related to Figure 3.

(A) TEM images of the blastopore at stage 18. Scale bar = 2 µm.
(B-C) Magnified view of the wide (B) or narrow gap (C) in areas enclosed by green and magenta boxes, respectively, in (A). Scale bars = 100 nm.
(D) The blastopore length in the slit long axis direction from St.14 to St.Ex. Blue line, mean.

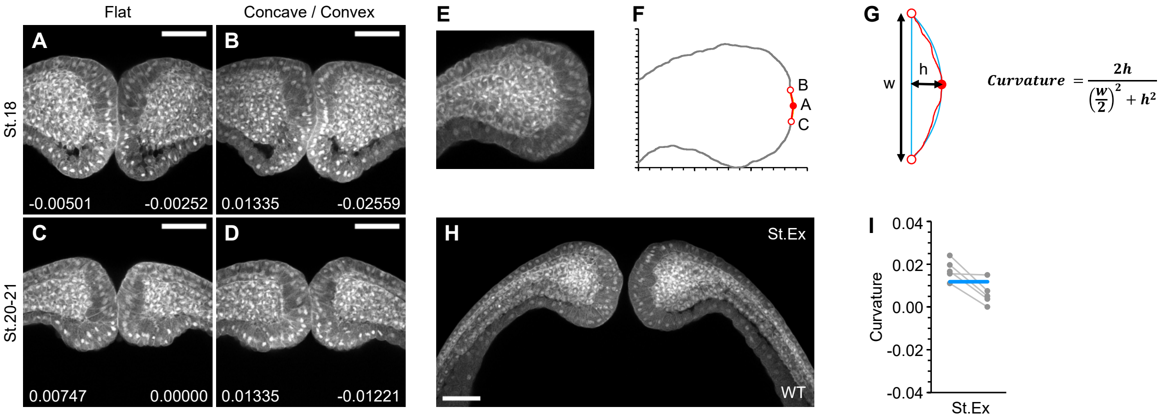

Figure S5. Measurement of CBC contact surface curvature, related to Figure 4.

(A-D) Representative images of flat/flat and concave/convex contact surfaces of the CBCs at stage 18. The lower left and lower right values indicate the curvature of the left and right CBC, respectively. Scale bars = 100 μm.
(E-F) An example of curvature analysis. The outline of CBC in (E) was extracted in (F), after which two points (red circle) ±32 μm apart vertically from the center of the slit (red dot) were set on the CBC outline.
(G) Curvature of the CBC contact surface (red curve) was approximated as the curvature of the blue arc. w = 64 μm.
(H) Embryos injected with fixative solution into the archenteron at the excretion stage. Representative coronal section cleared with BABB. Scale bar = 100 μm.
(I) Curvature of the CBC contact surface (n = 5). Left and right columns show CBC curvature on the left and right side, respectively. Gray lines correspond to the CBC of the same embryo. Blue line; mean.

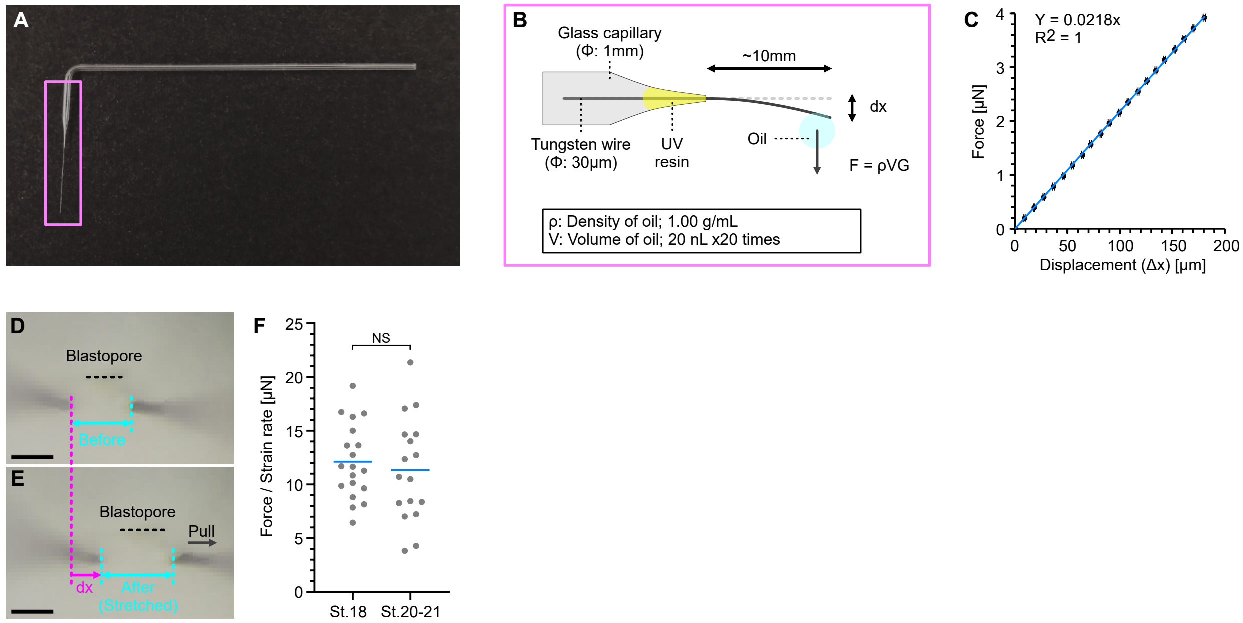

Figure S6. Calibration of force measurement probe, related to Figure 4.

(A) Image of force measurement probe.
(B) Schematic of force measurement probe in the area enclosed by the magenta square in (A). Relationship between strain and stress was estimated by repeatedly adding silicone oil to the tip of the measuring probe.
(C) Force-displacement curve of the probe showed linearity in at least the 0-4 μN range (N = 6). Blue dotted line, linear approximation of the data; error bars, ±SD.
(D, E) Schematic of CBC stiffness measurement. Scale bars = 100 μm.
(F) Force/strain of CBCs at St.18 (n = 19) and St.20-21 (n = 17). Blue line, mean. n = number of animals used. N = other data points.

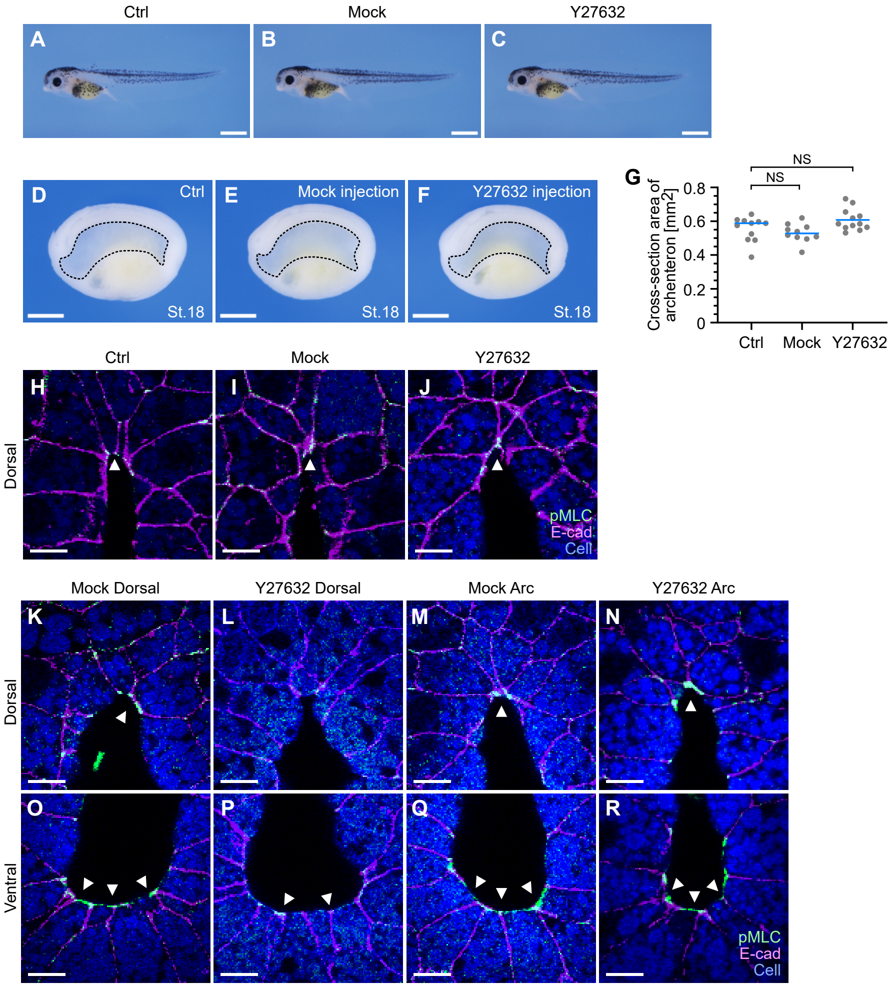

Figure S7. Effect of Y27632 injection on developmental and dorsal pMLC, related to Figure 6.

(A-C) No obvious developmental abnormalities were observed in tadpoles after injection of mock (n = 24/25) or Y27632 (n = 28/29) at stage 18. Ctrl, no injection (n = 31/31). Scale bars = 1mm.
(D-F) Representative sagittal section of control embryos (D), Mock-injected embryos (E), and Y27632-injected embryos (F) at stage 18. The black dotted line outlines the archenteron. Scale bars = 500 μm.
(G) Cross-section area of the archenteron in (D-F): Control embryos (n = 11), mock-injected embryos (n = 10), Y27632-injected embryos (n = 12). Blue lines, mean.
(H-J) Representative transverse section of the dorsal end of the blastopore in immunostained embryos. Embryos were fixed 10 minutes after injection of mock (I) or Y27632 (J). Ctrl, no injection. Green, pMLC; magenta, E-cadherin; blue, cells; white arrowhead, pMLC signals. Scale bars = 10 μm.
(K-R) Immunostaining of the blastopore using anti-pMLC and anti-E-cadherin antibodies. Cells were labelled by injection of 10 kDa Alexa Fluor 647-dextran. Representative images of a transverse section of the blastopore cleared with BABB at stage 18 are shown. Embryos underwent mock injection or injection of Y27632 into the archenteron (K, L, O, P) or the blastopore dorsal region (M, N, Q, R) and fixed after 10 min. Green, pMLC; magenta, e-cadherin; blue, cells; arrowheads, pMLC signals. Scale bars = 10 μm. n = number of animals used.
